# Approaches to economic evaluations of complex interventions in thailand: a systematic review: Author

**DOI:** 10.1186/s12889-025-25486-y

**Published:** 2025-11-25

**Authors:** Luxzup Wattanasukchai, Yuejiao Duan, Nicola McMeekin, Kathleen Boyd, Olivia Wu

**Affiliations:** 1 Health Economics and Health Technology Assessment (HEHTA), School of Health and Wellbeing, University of Glasgow, Glasgow, United Kingdom; 2 Clinical Epidemiology Unit, Faculty of Medicine, Khon Kaen University, Khon Kaen, Thailand

**Keywords:** Complex health intervention, Cost benefit analysis, Cost effectiveness analysis, Cost utility analysis, Health policy, Methodological approaches, Public health intervention, Thai

## Abstract

**Background:**

Complex interventions (CIs) are multifaceted health interventions that incorporate health, behavioural, social, organisational, and policy modifications at both individual and population levels. Therefore, evaluating the trade-offs between costs and benefits of CIs is challenging. In Thailand, CIs are increasingly being adopted in different settings and contexts. This study aims to synthesise the evidence on methodological approaches employed in economic evaluation of CIs within the Thai context.

**Methods:**

A systematic review of health economic evaluations of CIs in Thailand was conducted. Four databases (EMBASE, MEDLINE, EconLit, and HiTAP) were searched between January 2008 and July 2024. Studies were included if they met the United Kingdom Medical Research Council (MRC) definition for CIs (i.e., multicomponent design, behavioural targeting, expertise and skill requirements, multi-targeted group setting, and flexibility) and reported economic evaluations (cost-benefit, cost-consequence, cost-effectiveness, cost-utility, or cost-minimisation analyses) in Thailand. Non-English language and non-human studies were excluded. Relevant data on intervention types, methodological characteristics, and key findings were extracted and a narrative synthesis was carried out. The quality of included studies was assessed using the CHEERS checklist.

**Results:**

Sixty studies met the eligible criteria, categorised into screening programmes (33%), vaccination programmes (30%), other public health interventions (22%), and treatment and rehabilitation interventions (15%). Model-based cost-utility analysis from a societal perspective with lifetime horizon was the most frequently reported approach, particularly in screening programmes and treatment and rehabilitation interventions. while mid- and short-term horizons were more common for public health and treatment interventions, respectively. None explicitly identified interventions as CIs. However, alignment with MRC guidance was partial with limited use of designs capturing multidirectional, multisector effects, minimal stakeholder engagement, and underuse of advanced modelling techniques. Cost-benefit and cost-consequence analyses were underutilised. Most studies fulfilled the CHEERS criteria, but inconsistent reporting and weak stakeholder involvement indicated methodological gaps.

**Conclusion:**

Economic evaluations of CIs in Thailand have expanded since 2008. Partially adherence to the MRC framework, important gaps remain in comprehensive perspectives, early integration of economic considerations, and the use of advanced modelling techniques. Addressing these areas could enhance the robustness and policy relevance of future evaluations.

**Trial registration:**

CRD42024542012.

**Supplementary Information:**

The online version contains supplementary material available at 10.1186/s12889-025-25486-y.

## Introduction

Complex interventions (CIs) are multifaceted health interventions that typically involve various components targeting different aspects of a health issue [1]. CIs aim to tackle health challenges influenced by multiconnected factors at both individual and population levels [2]. These interventions often involve behavioural, social, organisational, and policy changes [3]. Initially introduced by the United Kingdom Medical Research Council (MRC) in 2000 [4], a framework for CIs was developed to assist researchers in identifying and implementing appropriate methodologies. This framework aims to improve the quality of research focused on developing and evaluating randomised controlled trials (RCTs). In 2008, the MRC formally defined CIs and updated its guidance to include a wider range of health interventions beyond RCTs [5]. The concept of CIs in health has evolved over a few decades, the MRC announcing the updated concept and its implementation in assessment in 2023 reflects advancements in the understanding and implementation [6]. CIs often require contextual adaptation to ensure effectiveness across diverse populations. However, evaluating their impacts poses significant challenges due to their inherent complexity and numerous influencing factors involved. These interventions typically require long-term implementation and demand collaboration among diverse stakeholders, including healthcare providers, policymakers, community organisations, and affected individuals, to achieve meaningful and sustainable improvements in health outcomes. Examples of CIs include robotic surgery implementation [7], smoking cessation programmes [8], and holistic community care initiatives [9].

One key challenge in assessing CIs is the need for a broader analytical scope that can accurately capture the full range of associated costs and benefits. In contrast to non-CIs, which involve a single, well-defined component to achieve a specific health outcome, that can be assessed through standard instructions and reproducible methods. The complexity of CIs demands a more comprehensive framework due to their multifaceted nature and the potential for externalities across multiple levels [10, 11]. The MRC defines complex interventions as those characterised by multiple interacting components, diverse behavioural outcomes, and varying demands on the expertise and skills of both implementers and participants. Complexity may also arise from the breadth of target populations, settings, or organisational levels involved, as well as from the degree of flexibility permitted in the delivery or adaptation of the intervention’s elements [8]. For example, an intervention aimed at reducing alcohol consumption may not only address alcohol-related issues such as mental health disorders, hepatitis, cirrhosis, and gastrointestinal bleeding, but also improve the quality of life for families and caregivers. Additionally, this intervention could contribute to decreased morbidity and mortality from alcohol-related road injuries [12]. The effects of these interventions should be considered as a nonlinear evaluation requiring carefully measurement of both costs and outcomes within a broader evaluative framework.

Most health interventions, especially those in the public health sector, involve complex interactions and require a deep understanding of health problems that extend beyond individual-level concerns, often impacting entire communities or populations. Consequently, the evaluation of multicomponent interventions presents methodological challenges due to their complexity and the need for standardised evaluative frameworks [13]. Economic evaluation (EE) plays a crucial role in assessing the efficiency of CIs by focusing on health resource utilisation and long-term benefits, rather than solely on intermediate outcomes or process measures [14].

In Thailand, the application of EE is growing with the increasing emergence of public health interventions and CIs. The MRC recommends incorporating EE early and consistently throughout the CI research process to assess the comparative costs and consequences for affected populations [6]. In the Thai context, however, there is limited attention to EE of CIs and existing evidence does not sufficiently identify the methodological approaches used [15]. As CIs are increasingly adopted in various Thai settings and context, it is crucial to understand the current evidence related to EE of CIs in the country. This study aims to comprehensively summarise the methodological approaches employed in the EE of CIs within Thailand, thereby informing the development and refinement of future evaluations.

## Methods

This systematic review examines EEs of complex interventions, which involve a comparative analysis of costs and benefits, including cost-benefit analysis, cost-consequence analysis, cost-effectiveness analysis, cost-minimisation analysis, and cost-utility analysis, conducted in the context of Thailand. The review adhered to the Preferred Reporting Items for Systematic Reviews and Meta-Analyses (PRISMA) 2020 guidelines [16] and was registered with the International Prospective Register of Systematic Reviews (PROSPERO) (CRD42024542012).

### Search strategy

A comprehensive search for EEs of CIs was conducted in three electronic databases: EMBASE, MEDLINE, and EconLit. Additionally, the Health Intervention and Technology Assessment Program (HiTAP) database was manually searched for additional studies. The search terms, which were developed following the PICOS criteria (Supplement Table 1), included “complex intervention”, “economic evaluation,” “cost-minimisation analysis,” “cost-effectiveness analysis,” “cost-benefit analysis,” “cost-utility analysis,” “cost-consequence analysis,” and “Thailand”.

The search results were confined to the period from January 2008 to July 2024. The year 2008 was selected as the starting point for two main reasons. First, the MRC introduced its definition and guidance on evaluating CIs in 2008 [5], providing a conceptual framework that has since shaped methodological standards. Second, Thailand published its first national health technology assessment guidance in the same year, outlining how to conduct economic evaluations to inform policy decisions [17]. Together, these milestones mark a pivotal point in the development and application of economic evaluation methods in health care. Only peer reviewed EE published in English, involving human subjects, were included. Cost-only studies and abstracts without full-text articles were excluded. Detailed search terms and eligibility filters are presented in Supplement Table 2.

### Eligibility criteria and study selection

We retrieved articles following the PRISMA guidelines [16]. Titles and abstracts of all retrieved electronic citations were screened for eligibility by one reviewer (LW). To ensure accuracy, a random sample comprising 20% of titles and abstracts was independently assessed by a second reviewer (YD). Following Cochrane recommendations [18], if discrepancies in inclusion decisions exceeded 20% within this process, independent re-screening of all records would have been undertaken. A similar procedure was applied during the full-text screening of potentially eligible studies. Any disagreements between reviewers were resolved through discussion with additional reviewers (OW and KB). Inter-rater reliability was assessed and reported using Cohen’s kappa coefficient [19]. Finally, all included studies were re-checked by OW and KB to confirm adherence to the inclusion criteria. The included studies encompassed various forms of EE (i.e., cost-benefit analysis (CBA), cost-effectiveness analysis (CEA), cost consequence analysis (CCA), cost-minimisation analysis, and cost-utility analysis (CUA)). The studies were included when they met at least one of the MRC’s five criteria for CIs. The criteria included (1) multicomponent design, (2) behavioural targeting, (3) expertise and skill requirements, (4) multi-targeted group setting or level, and (5) flexibility [6]. Studies were excluded if they meet exclusion criteria (e.g., abstract only, non-human studies, non-EE). (Supplement Table 3)

### Data extraction

LW extracted information from all included studies using data extraction forms in Microsoft Excel. YD independently verified 20% of the included articles. If discrepancies in the extracted data had exceeded 20% within this process, full independent re-extraction of all included articles would have been undertaken. Any disagreements were resolved through discussion with OW and KB. Extracted data included (1) general information (e.g., title, authors, publication year, location), (2) characteristics of interventions and comparators, (3) methodological characteristics (e.g., type of economics evaluation, measurement and sources of costs, measurement and sources of outcomes, and 4) bases case analysis and uncertainty analysis.

### Quality assessment

The quality of the included studies was assessed using the Consolidated Health Economic Evaluation Reporting Standards statement (CHEERS) 2022 checklist [20]. With the intention of assessing overall reporting quality, the included studies were assigned “yes” if the requirement from the checklist was fulfilled, “partially yes” when partially fulfilled, “no” when no or insufficient information was available, and “NA” when not applicable was reported. Although the CHEERS checklist is not designed as a quality assessment instrument, the application of the CHEERS checklist has been adapted in several published systematic reviews [21]. Two reviewers independently assessed quality of the included study. Disagreements were resolved through consensus.

### Data synthesis

A narrative synthesis of all included studies was provided. This description was included tables, summarising key study details, including study design, participant characteristics, details of interventions and comparators, reported outcome measures, and quality assessments. All included studies were evaluated using the MRC framework for evaluating CIs [8]. All data were collected and analysed using Microsoft Excel.

## Results

### Study selection

**Fig. 1:**
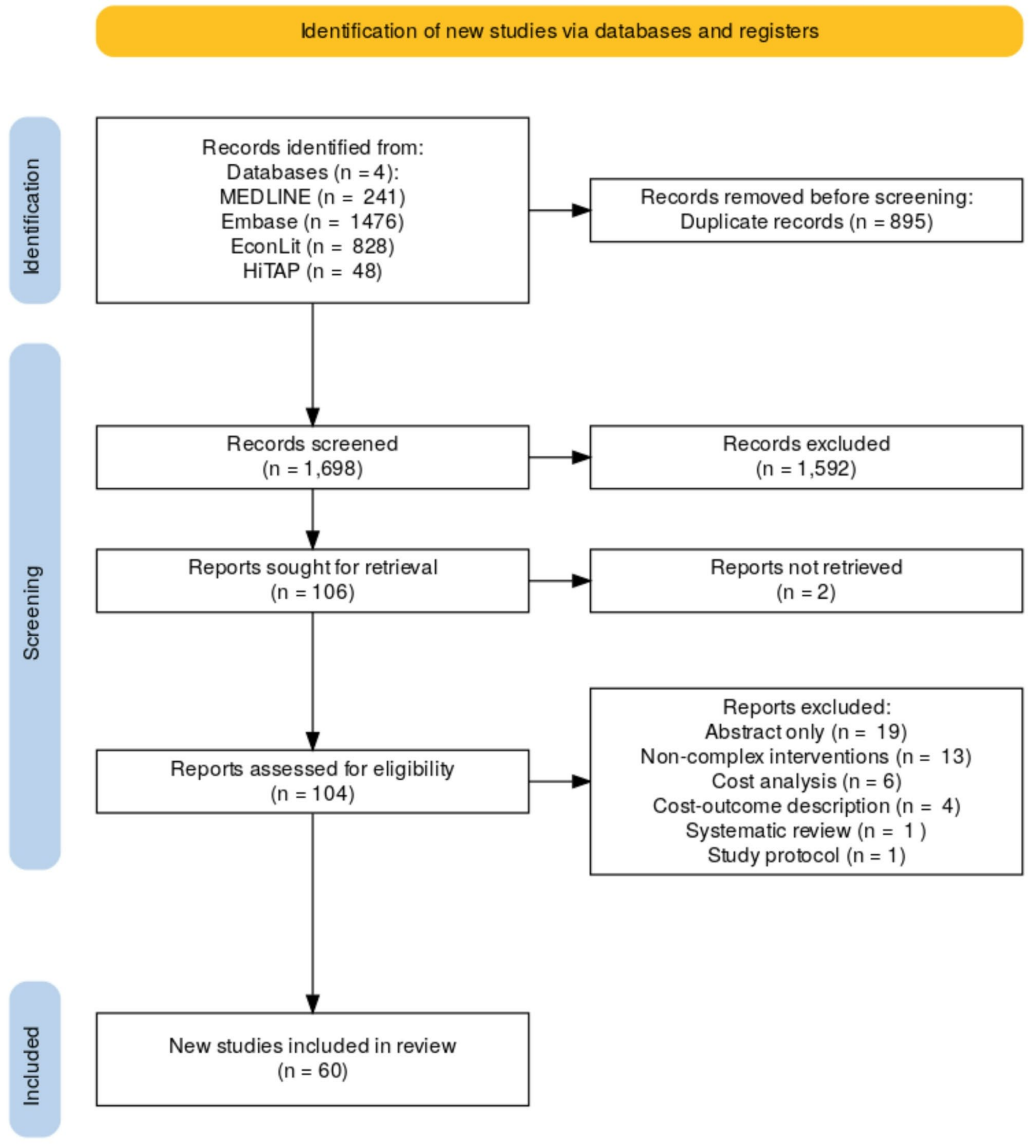
PRISMA flow diagram

The study selection process is illustrated in the PRISMA diagram (Fig. 1). A total of 2,593 records were identified through the electronic databases. After removing 895 duplicates, 1,698 articles remained for screening. Of these, 1,592 articles were excluded based on their titles and abstracts because they did not meet eligibility criteria. Subsequently, 104 full-text articles were assessed for selection eligibility. Exclusion reasons were related to reports study designs, (i.e., non-EEs), intervention type and publication format. Ultimately, 60 EE studies were deemed suitable for inclusion in the systematic review [22–81]. The inter-reviewer disagreement was 19.4% (kappa 0.81), which corresponds to minimal disagreement and therefore no further double screening was required.

### Characteristics of the included studies

A total of 60 studies, published between 2008 and 2024, were included in the systematic review [22–81] (Table 1). However, there was an increase in publications occurred in the last two years, accounting for nearly one-quarter of included studies [23, 24, 28, 30, 36, 40, 44, 49, 50, 55, 56, 64, 65, 71]. 75% of the included articles were identified as CUA based on quality-adjusted life-years (QALYs) (*n* = 38) [23, 26, 28, 31–40, 42–45, 49, 51–63, 65, 66, 73, 75, 76, 78, 81] and disability-adjusted life-years (DALYs) (*n* = 7) [25, 27, 29, 41, 47, 69, 77]. Over 90% performed model-based EE [22–31, 34–55, 57–66, 68–71, 73–81], which consisted of a combination of decision tree and Markov models [23, 26, 34, 37, 38, 41, 42, 44, 52, 53, 57, 60, 63, 65, 74–76, 78], Markov model [28, 31, 39, 43, 49, 51, 54, 55, 58, 59, 61, 64, 66, 73], decision tree model [22, 25, 27, 29, 30, 35, 40, 46–48, 71, 77, 79, 81], dynamic transmission model [24, 41, 69, 70, 80], and epidemiological model [68]. Two model-based studies did not provide information about their models [50, 62], while five remaining studies designed EE alongside clinical trials (EEACTs) [32, 33, 56, 67, 72].

**Table 1:** Summary of the included studies

	Type of interventions				Total		
	Public health interventions*	Screening programmes	Vaccination programmes	Treatment and rehabilitation interventions			
N (%)	13 (22)	20 (33)	18 (30)	9 (15)	60 (100)		
Year of publication							
2008–2012	1 (8)	4 (20)	5 (28)	3 (33)	13 (22)		
2013–2017	3 (23)	7 (35)	3 (17)	4 (44)	17 (28)		
2018–2022	3 (23)	5 (25)	7 (39)	1 (11)	16 (27)		
2023–2024	6 (46)	4 (20)	3 (17)	1 (11)	14 (23)		
Type of economic evaluation							
Cost-utility analysis	5 (38)	17 (85)	15 (83)	8 (89)	45 (75)		
Cost-effectiveness analysis	7 (54)	1 (5)	4 (22)	1 (11)	13 (22)		
Cost- benefit analysis	3 (23)	1 (5)	0 (0)	0 (0)	4 (7)		
Cost-minimisation analysis	0 (0)	0 (0)	0 (0)	0 (0)	0 (0)		
Cost-consequence analysis	0 (0)	0 (0)	0 (0)	0 (0)	0 (0)		
More than one economic evaluation framework	2 (15)	0 (0)	1 (6)	0 (0)	3 (5)		
Economic modelling							
Combination model of decision tree and Markov models	2 (15)	11 (55)	6 (33)	0 (0)	19 (32)		
Markov model	2 (15)	4 (20)	4 (22)	4 (44)	14 (23)		
Decision tree	6 (46)	4 (20)	3 (17)	1 (11)	14 (23)		
EEACTs	0 (0)	1 (5)	0 (0)	4 (44)	5 (8)		
Dynamic transmission model	1 (8)	0 (0)	4 (22)	0 (0)	5 (8)		
Unidentified model type	1 (8)	0 (0)	1 (6)	0 (0)	2 (3)		
Epidemiological model	1 (8)	0 (0)	0 (0)	0 (0)	1 (2)		
Analytical time horizon							
≤ 1 year	3 (23)	2 (10)	7 (39)	4 (44)	16 (27)		
Up to 5 years	2 (15)	0 (0)	3 (17)	0 (0)	5 (8)		
6–10 years	2 (15)	0 (0)	0 (0)	0 (0)	2 (3)		
≥ 20 years	2 (15)	0 (0)	0 (0)	0 (0)	2 (3)		
Lifetime	5 (38)	18 (90)	7 (39)	5 (56)	35 (58)		
Not reported	0 (0)	0 (0)	1 (6)	0 (0)	1 (2)		
More than one analytical time horizon	1 (8)	0 (0)	0 (0)	0 (0)	1 (2)		
Study perspective							
Societal	7 (54)	18 (90)	11 (61)	6 (67)	42 (70)		
Health care providers/hospital	2 (15)	5 (25)	4 (22)	1 (11)	12 (20)		
Government	0 (0)	2 (10)	2 (11)	3 (33)	7 (12)		
Health system	4 (31)	0 (0)	= 3 (17)	3 (33)	10 (17)		
Patients	1 (8)	0 (0)	0 (0)	0 (0)	1 (2)		
Not reported	1 (8)	1 (0)	0 (0)	0 (0)	2 (3)		
More than one perspective	2 (15)	6 (30)	3 (17)	3 (33)	14 (23)		
Annual Discounting							
3% for both costs and outcomes	8 (62)	16 (80)	13 (72)	6 (67)	43 (72)		
3.25% for costs	1 (8)	0 (0)	0 (0)	0 (0)	1 (2)		
5% for both costs and outcomes	1 (8)	0 (0)	0 (0)	0 (0)	1 (2)		
Non discount	1 (8)	0 (0)	2 (11)	0 (0)	3 (5)		
Not reported	3 (23)	4 (20)	3 (17)	3 (33)	13 (22)		
More than one discount rate	1 (8)	0 (0)	0 (0)	0 (0)	1 (2)		

*EEACT* economic evaluation alongside clinical trial

*excluding screening and vaccination programmes

More than half of the included studies adopted a lifetime horizon [24, 26, 27, 29, 31, 34, 36–44, 49, 51–55, 57, 59–61, 63, 65, 66, 73–76, 78, 79, 81] and two studies used horizons longer than 20 years [28, 68], while seven applied shorter timeframes ranging from 5 to 10 year [30, 45–48, 50, 58]. Sixteen studies used a short-term horizon of one year or less [22–24, 32, 33, 35, 56, 62, 64, 67, 69–72, 77, 80] and one did not report the time horizon [25].

Regarding discounting, 72% applied an annual rate of 3% for costs and health outcomes, consistent with the Thai guidance [24–29, 31, 34, 36–39, 41–45, 47, 49, 51–55, 57–68, 73–78, 81]. Two studies did not apply discounting due to the shot time horizons (≤ 1 year) [35, 70], while one employed a nine-year horizon without discounting [50]. Other variations included, a 3.25% discount for costs only [46] and 5% rate for both costs and outcomes [68]. In contrast, thirteen studies did not report discount rate [22, 23, 30, 32, 33, 40, 48, 56, 69, 71, 72, 79, 80].

In total, 74 analytical perspectives were identified across the 60 studies [22–81]. Fourteen studies adopted dual perspectives (e.g., the government and societal perspectives, and the health care providers and patients’ perspectives) [31, 32, 37, 43, 47, 48, 54, 58, 60, 61, 65, 68, 79, 81]. The most common perspective was societal (*n* = 42) [24, 26, 28, 31–41, 43–47, 49, 52–63, 65, 66, 68, 75–81], followed by health care providers or hospital [22, 23, 43, 47, 48, 54, 64, 65, 67, 71, 73, 81], government [32, 42, 51, 60, 69, 70, 79], and health system perspectives [25, 27, 29–31, 37, 58, 61, 68, 74].

### Types of complex interventions

The interventions were classified into four types: (1) screening programme (*n* = 20) [22, 34, 36, 39, 40, 43, 44, 52–54, 57, 60, 63, 65, 66, 72, 75, 78, 79, 81], (2) vaccination programmes (*n* = 18) [23, 25, 26, 35, 37, 38, 41, 42, 45, 47, 49, 58, 62, 64, 69, 70, 73, 80], (3) other public health interventions (excluding screening and vaccination programmes) (*n* = 13) [24, 27, 28, 30, 46, 48, 50, 55, 68, 71, 74, 76, 77], and (4) treatment and rehabilitation interventions (*n* = 9) [29, 31–33, 51, 56, 59, 61, 67].

#### Screening programmes

Screening programmes [22, 34, 36, 39, 40, 43, 44, 52–54, 57, 60, 63, 65, 66, 72, 75, 78, 79, 81] aimed to identify individuals at risk of developing disease or condition before symptom onset to reduce morbidity and mortality. These included both individual-level screenings [40, 44, 53, 57, 60, 63, 65, 78] and population-based programmes [22, 34, 36, 39, 43, 54, 66, 72, 75, 79, 81]. In Thailand, targeted conditions included various cancers [36, 39, 40, 44, 52, 54, 60], other diseases [22, 34, 43, 53, 65, 66, 72, 75, 79, 81], and pharmacogenetic risk [57, 63, 78] (Table 2).

**Table 2:** Summary of characteristics of the screening programmes

No	Author, year	Population	Intervention(s) and comparator(s)	Complexity of interventions	Methods
1	Tengtrisorn [72], 2009	Primary school children	Interventions: 1. Active visual screening by medical personnel 2. Visual Screening by trained teaches Comparator: Walk-in patients at hospital	- Integrated school-based screening with referral system. - Implementation required budget and resource allocations from schools, local government, Ministry of Education, Ministry of Public Health, and health system payers.	EE type: CEA Economic modelling: EEACT Perspective: NA Time horizon: 1 year Discounting: NA
2	Leelukkanaveera [43], 2010	Patient with unknow HIV status	Intervention: Provider-initiated HIV counselling and testing Comparator: Current practice	- Integrated testing, counselling, referral, and treatment system. - Required trained counsellors to increase test acceptance and linkage to care.	EE type: CUA Economic modelling: Markov model Perspective: Health care providers and societal Time horizon: Lifetime Discounting: 3%*
3	Praditsitthikorn [54], 2011	Thai women aged ≥ 15 years old	Interventions: National cervical cancer prevention and control strategies Comparator: Do-nothing	- National-level integrated multiple prevention strategies with treatment and referral system across individual, community, hospital, local- and national- health organisations, and health system payers. - Flexible policy for difference target groups and policy strategies. - National-level integrated prevention strategies with treatment and referral across individual, community, hospital, local and national health organisations, and healthcare payers. - Flexible policy tailored to different target groups.	EE type: CUA Economic modelling: Markov models Perspectives: Health care providers and societal Time horizon: Lifetime Discounting: 3%*
4	Kingkaew [34], 2012	Thai postmenopausal women	Interventions: Combination of OA screening and treatment strategies Comparator: Do-nothing	- Integrated screening and treatment strategies across communities, hospitals, and healthcare payers.	EE type: CUA Economic modelling: Combination model of decision tree and Markov models Perspective: Societal Time horizon: Lifetime Discounting: 3%*
5	Rattanavipapong [57], 2013	Newly diagnosed epilepsy or neuropathic pain patients	Interventions: 1. Universal HLA-B*15:02 screening 2. Alternative drug prescription Comparator: Current practice	- National-level implementation across hospitals, genetic laboratories, and healthcare payers to establish testing and referral pathways.	EE type: CUA Economic modelling: Combination model of decision tree and Markov models Perspective: Societal Time horizon: Lifetime Discounting: 3%*
6	Sangmala [60], 2014	Patients with HBsAg positive or chronic HBV carriers without antiviral treatment	Interventions: Multimodalities HCC surveillance programmes Comparator: Current practice	- National-level multimodality screening, treatment, and referral system across the healthcare system. - Required radiologists for US.	EE type: CUA Economic modelling: Combination model of decision tree and Markov Perspectives: Government and societal Time horizon: Lifetime Discounting: 3%*
7	Saokaew [63], 2014	Patients who received allopurinol for preventing or treating gout, hyperuricemia, or tophaceous gout	Intervention: Universal HLA-B*5801 testing Comparator: Current practice	- National-level implementation across hospitals, genetic laboratories, and payers to establish testing and referral pathways.	EE type: CUA Economic modelling: Combination model of decision tree and Markov models Perspective: Societal Time horizon: Lifetime Discounting: 3%*
8	Srisubat [66], 2014	Diabetic patients with normotension	Intervention: National annual microalbuminuria screening Comparator: Do-nothing	- National-level implementation across the healthcare system.	EE type: CUA Economic modelling: Markov model Perspective: Societal Time horizon: Lifetime Discounting: 3%*
9	Thiboonboon [75], 2015	Thai infants	Intervention: Expanded neonatal screening for inborn errors of metabolism Comparator: Current practice	- National-level implementation across hospitals, laboratories, and healthcare payers.	EE type: CUA Economic modelling: Combination model of decision tree and Markov models Perspective: Societal Time horizon: Lifetime Discounting: 3%*
10	Wongwai [81], 2017	Thai infants	Intervention: Digital photographic ROP screening using the RetCam Comparator: Current practice	- National-level telemedicine screening and referral system across hospitals. - Required trained technicians and ophthalmologists. - Adaptable to different settings.	EE type: CUA Economic modelling: Decision tree model Perspectives: Health care providers and societal Time horizon: Lifetime Discounting: 3%*
11	Phisalprapa [53], 2017	Metabolic syndrome patients	Intervention: US screening for non-alcoholic fatty liver disease with intensive weight reduction Comparator: Do-nothing	- Multi-level screening and referral system involving individuals, communities, and hospitals. - Required radiologists, clinicians, dietitians, and counsellors. - Active patient behaviour and lifestyle modifications.	EE type: CUA Economic modelling: Combination model of decision tree and Markov models Perspective: Societal Time horizon: Lifetime Discounting: 3%*
12	Kumdee [39], 2018	Thai adults aged > 40 years olds	Intervention: Oral cancer screening policies Comparator: Do-nothing	- National-level interactive screening and referral system across communities and healthcare settings with varying examiners. - Required trained dental professionals - Adaptable to different settings.	EE type: CUA Economic modelling: Markov model Perspective: Societal Time horizon: Lifetime Discounting: 3%*
13	Phisalprapa [52], 2019	Asymptomatic Thai adults with average-risk of colorectal cancer	Interventions: Colorectal cancer screening policies Comparator: Do-nothing	- National-level implementation of screening, treatment, and referral across communities, hospitals, and healthcare payers.	EE type: CUA Economic modelling: Combination model of decision tree and Markov models Perspective: Societal Time horizon: Lifetime Discounting: 3%*
14	Wanapirak [79], 2019	Pregnant women	Interventions: Foetal Down syndrome screening strategies Comparators: Do-nothing	- National-level integrated multimodality screening and referral system. - Benefits for maternal and child health.	EE type: CBA Economic modelling: Decision tree model Perspectives: Government and societal Time horizon: Lifetime Discounting: 3%*
15	Bierhoff [22], 2021	Pregnant women at refugee sites on the Thai–Myanmar border	Interventions: Screening and management strategies of mother-to-child transmission of HBV Comparator: HBV vaccination without maternal screening	- Integrated screening, vaccination, and treatment involving Thai and international organisations in refugee settings. - Benefits for maternal and child health - Implementation with equity concerns.	EE type: CEA Economic modelling: Decision tree model Perspective: Health care providers Time horizon: 1 year Discounting: 3%*
16	Turongkaravee [78], 2022	Newly diagnosed HIV patients who received ART and treatment for opportunistic	Interventions: Pharmacogenetics testing strategies Comparator: Current practice	- National-level integrated strategies for HIV testing, bioinformatics, counselling, treatment, and referral. - Required trained counsellors to increase test acceptance and linkage to care.	EE type: CUA Economic modelling: Combination model of decision tree and Markov models Perspective: Societal Time horizon: Lifetime Discounting: 3%*
17	Kositamongkol [36], 2023	Unvaccinated Thai women for HPV vaccines	Interventions: Cervical cancer screening programmes Comparator: Do-nothing	- National-level integrated strategies for screening, treatment, and referral across home and hospital settings.	EE type: CUA Economic modelling: Combination model of decision tree and Markov models Perspective: Societal Time horizon: Lifetime Discounting: 3%*
18	Laopachee [40], 2023	High-risk CCA population in Northern Thailand	Intervention: Upper abdominal US surveillance Comparator: Do-nothing	- Integrated interventions for surveillance, diagnosis, referral, and treatment in communities and hospitals. - Required trained physicians or radiologists.	EE type: CUA Economic modelling: Decision tree model Perspective: Societal Time horizon: Lifetime Discounting: 3%*
19	Lertwilaiwittaya [44], 2023	Breast cancer patients and high-risk relatives	Intervention: BRCA1/BRCA2 genetic testing Comparator: Do-nothing	- National-level screening and referral system for patients and relatives across providers, laboratories, hospitals, government organisations, and healthcare payers.	EE type: CUA Economic modelling: Combination model of decision tree and Markov models Perspective: Societal Time horizon: Lifetime Discounting: 3%*
20	Srisubat [65], 2023	T2D patients without previously screened for DR	Intervention: Annually DR screening using deep learning Comparator: Screening by non-physician human graders	- National-level implementation of integrated screening and treatment by trained technicians and ophthalmologists.	EE type: CUA Economic modelling: Combination model of decision tree and Markov models Perspectives: Health care providers and societal Time horizon: Lifetime Discounting: 3%*

*ART* anti-retroviral therapy, *BRCA* breast cancer gene, *CBA* cost-benefit analysis, *CCA* cholangiocarcinoma, *CEA* cost-effectiveness analysis, *CUA* cost-utility analysis, *DR* diabetic retinopathy, *EE* economic evaluation, *EEACT* economic evaluation alongside clinical trial, *HBV* hepatitis B virus, *HBsAg* hepatitis B s-Antigen, *HCC* hepatocellular carcinoma, *HIV* human immunodeficiency virus, *HLA-B* major histocompatibility complex, class I, B, *HPV* human papilloma virus, *NA* not assessed, *OA* osteoarthritis, *ROP* retinopathy of prematurity, *T2D* Type 2 diabetes, *US* ultrasonography

*both costs and outcomes

CUA was the dominant method [34, 36, 39, 40, 43, 44, 52–54, 57, 60, 63, 65, 66, 75, 78, 81], highlighting the emphasis on quantifying health outcomes in terms of QALY. A combination approaches integrating decision trees and Markov models were frequently used [34, 36, 44, 52, 53, 57, 60, 63, 65, 75, 78]. Most studies applied lifetime horizon [34, 36, 39, 43, 44, 52–54, 57, 60, 63, 65, 66, 75, 78, 81], reflecting the long-term implications and inherent complexity of these interventions.

#### Vaccination programmes

Vaccination programmes [23, 25, 26, 35, 37, 38, 41, 42, 45, 47, 49, 58, 62, 64, 69, 70, 73, 80] represent coordinated public health initiatives aimed at immunising populations against infectious diseases. These programmes focused on diseases such as rotavirus [25, 45, 47, 58, 62], COVID-19 [64, 70, 80], pneumococcal [26, 38, 49], influenza [35, 69], and others [23, 37, 41, 42, 73] (Table 3). Evaluating these programmes is complex that require consideration of substantial costs for vaccine planning, administration, storage, and distribution, as well as factors such as long-term protective efficacy, potential adverse effects, and the impact of herd immunity.

**Table 3:** Summary of characteristics of the vaccination programmes

No	Author, year	Population	Intervention(s) and comparator(s)	Complexity of interventions	Methods
1	Chotivitayatarakorn [25], 2010	Thai infants	Interventions: National rotavirus vaccinations Comparator: No vaccination programme	- National-level implementation involving individuals, communities, local health providers, healthcare payers, and national health authorities across supply chains, delivery, distribution, administration, and monitoring.	EE type: CEA and CUA Economic modelling: Decision tree model Perspective: Health system Time horizon: NA Discounting: 3%*
2	Lee [41], 2011	Dengue-naive infants aged ≤ 1 year old	Intervention: National dengue vaccination Comparator: No vaccination programme	- National-level implementation involving individuals, communities, local health providers, healthcare payers, and national health authorities across supply chains, delivery, distribution, administration, and monitoring.	EE type: CUA Economic modelling: Combination model of decision tree and Markov models Perspective: Societal, Time horizon: Lifetime Discounting: 3%*
3	Leelahavarong [42], 2011	1. Thai population aged 18 - 30 years olds 2. FSW 3. IDU 4. MSM 5. Military conscripts	Intervention: HIV vaccine combined with HIV prevention programmes: 1) free condoms 2) school-based education 3) anti-retroviral prophylaxis services for all pregnant women 4) diagnosis and treatment of STI 5) needle and syringe programme 6) screening blood products and donated organs for HIV. Comparator: HIV prevention programmes	- National-level integrated HIV vaccination and prevention strategies across communities, providers, payers, and authorities. - Vaccination strategies varied according to risk behaviours.	EE type: CUA Economic modelling: Combination model of decision tree and Markov models Perspective: Government Time horizon: Lifetime Discounting: 3%*
4	Muangchana [47], 2012	Thai children	Intervention: National rotavirus vaccinations Comparator: No vaccination programme	- National-level implementation involving individuals, communities, local health providers, healthcare payers, and national health authorities across supply chains, delivery, distribution, administration, and monitoring.	EE type: CUA Economic modelling: Decision tree model Perspectives: Health care providers and societal Time horizon: 5 years Discounting: 3%*
5	Termrungruanglert [73], 2012	Thai girls who never had sexual intercourse	Intervention: National HPV vaccination Comparator: No vaccination programme	- Integrated intervention across prevention, diagnosis, and treatment pathways. - National-level implementation involving individuals, communities, local health providers, healthcare payers, and national health authorities across supply chains, delivery, distribution, administration, and monitoring.	EE type: CUA Economic modelling: Markov model Perspective: Health care providers Time horizon: Lifetime Discounting: 3%*
6	Kulpeng [38], 2013	Thai infants	Intervention: National pneumococcal vaccinations Comparator: No vaccination programme	- National-level implementation involving individuals, communities, local health providers, healthcare payers, and national health authorities across supply chains, delivery, distribution, administration, and monitoring.	EE type: CUA Economic modelling: Combination model of decision tree and Markov models Perspective: Societal, Time horizon: Lifetime Discounting: 3%*
7	Kittikraisak [35], 2017	Healthy children aged ≤ 5 years old in Bangkok	Intervention: National influenza vaccinations Comparator: No vaccination programme	- National-level implementation involving individuals, communities, local health providers, healthcare payers, and national health authorities across supply chains, delivery, distribution, administration, and monitoring.	EE type: CUA Economic modelling: Decision tree model Perspective: Societal Time horizon: 1 year Discounting: No discounting
8	Kotirum [37], 2017	Thai children aged < 5 years old	Intervention: National Hib vaccination Comparator: No vaccination programme	- National-level implementation involving individuals, communities, local health providers, healthcare payers, and national health authorities across supply chains, delivery, distribution, administration, and monitoring.	EE type: CUA Economic modelling: Combination model of decision tree and Markov models Perspectives: Health system and societal Time horizon: Lifetime Discounting: 3%*
9	Dilokthornsakul [26], 2019	Thai infants aged < 6 months	Interventions: National pneumococcal vaccinations Comparator: No vaccination programme	- National-level implementation involving individuals, communities, local health providers, healthcare payers, and national health authorities across supply chains, delivery, distribution, administration, and monitoring.	EE type: CUA Economic modelling: Combination model of decision tree and Markov models Perspective: Societal, Time horizon: Lifetime Discounting: 3%*
10	Saokaew [62], 2019	Thai children aged < 5 years old	Interventions: National rotavirus vaccinations Comparator: No vaccination programme	- National-level implementation involving individuals, communities, local health providers, healthcare payers, and national health authorities across supply chains, delivery, distribution, administration, and monitoring.	EE type: CUA Economic modelling: NA Perspective: Societal Time horizon: 1 year Discounting: 3%*
11	Suphanchaimat [69], 2020	Thai prisoners	Intervention: Influenza vaccination policy Comparator: routine outbreak control	- Implementation of integrated outbreak control (surveillance, quarantine, and treatment) across prisoners, communities, local hospitals, and government (Ministry of Justice, and Ministry of Public Health)	EE type: CUA Economic modelling: Dynamic transmission model Perspective: Government Time horizon: 1 year Discounting: NA
12	Luangasanatip [45], 2021	Thai infants aged < 1 year old	Interventions: National rotavirus vaccinations Comparator: No vaccination programme	- National-level implementation involving individuals, communities, local health providers, healthcare payers, and national health authorities across supply chains, delivery, distribution, administration, and monitoring.	EE type: CUA Economic modelling: Dynamic transmission model Perspective: Societal Time horizon: 5 years Discounting: 3%*
13	Rochanathimoke [58], 2021	Thai children	Interventions: National rotavirus vaccinations Comparator: No vaccination programme	- National-level implementation involving individuals, communities, local health providers, healthcare payers, and national health authorities across supply chains, delivery, distribution, administration, and monitoring.	EE type: CUA Economic modelling: Markov model Perspectives: Health system and societal Time horizon: 5 years Discounting: 3%*
14	Suphanchaimat [70], 2021	Thai residences and migrants in Samut Sakorn province	Interventions: COVID-19 vaccination strategies Comparator: Do-nothing	- Implementation involved individuals, communities, local health providers, health care payers, and national health authorities across supply chains, delivery, distribution, administration, and monitoring. - Flexible policy for multi-population (residents, registered and unregistered migrants, healthcare workers) - Implementation with ethical and equity concerns	EE type: CEA Economic modelling: Dynamic transmission model Perspective: Government, Time horizon: 1 years Discounting: No discounting
15	Wang [80], 2022	1. High-risk elderly aged ≥ 65 years old 2. High-incidence group aged 20–39 years old	Intervention: COVID‑19 vaccinations Comparator: No vaccination programme	- National-level implementation involving individuals, communities, local health providers, healthcare payers, and national health authorities across supply chains, delivery, distribution, administration, and monitoring. - Flexible policy for multi-population - Implementation with ethical and equity concerns	EE type: CEA Economic modelling: Dynamic transmission model Perspective: Societal Time horizon: 1 year Discounting: NA
16	Botwright [23], 2023	Pregnant women	Intervention: National maternal pertussis vaccinations Comparator: Current practice	- National-level implementation involving local health providers, health care payers, and national health authorities across supply chains, delivery, distribution, administration, and monitoring. - Benefits for maternal and child health	EE type: CUA Economic modelling: Combination model of decision tree and Markov models Perspective: Health care providers Time horizon: 1 year Discounting: NA
17	Ngamprasertchai [49], 2023	Thai who were 1. healthy or with chronic health conditions 2. immunocompromised conditions (HIV infection, lymphoma, or generalised malignancy)	Interventions: National pneumococcal vaccinations Comparator: No vaccination programme	- National-level implementation involving individuals, communities, local health providers, healthcare payers, and national health authorities across supply chains, delivery, distribution, administration, and monitoring.	EE type: CUA Economic modelling: Markov model Perspective: Societal, Time horizon: Lifetime Discounting: 3%*
18	Sirison [64], 2023	Thai adults	Interventions: COVID-19 booster vaccinations Comparator: No vaccination programme	- National-level implementation involving individuals, communities, local health providers, healthcare payers, and national health authorities across supply chains, delivery, distribution, administration, and monitoring. - Implementation with equity concerns	EE type: CEA Economic modelling: Markov Perspective: Health care providers Time horizon: 170 days Discounting: 3%*

*CEA *cost-effectiveness analysis, *COVID-19* Coronavirus disease 2019: *CUA* cost-utility analysis, *EE* economic evaluation, *FSW* female sex worker, *Hib* Haemophilus influenzae type b, *HIV* human immunodeficiency virus, *HPV* human papilloma virus, *IDU* injecting drug user, *MSM* men who have sex with men, *NA* not assessed, *STI* sexually transmitted infection

*both costs and outcomes

In Thailand, vaccination programmes primarily relied on CUAs [23, 25, 26, 35, 37, 38, 41, 42, 45, 47, 49, 58, 62, 69, 73], while CEAs were less frequently employed [25, 64, 70, 80]. Common modelling approaches included decision tree and Markov combinations [23, 26, 37, 38, 41, 42] over a lifetime horizon [26, 37, 38, 41, 42, 49, 73], reflecting the long-term protective effects of vaccines. However, dynamic transmission models were adopted for evaluating the transmission of infectious diseases [45, 69, 70, 80]. The societal perspective [26, 35, 37, 38, 41, 45, 47, 49, 58, 62, 80] with 3% discounting [25, 26, 37, 38, 41, 42, 45, 47, 49, 58, 62, 64, 73] was the most frequently applied.

#### Public health interventions (excluding screening and vaccination programmes)

Other public health interventions [24, 27, 28, 30, 46, 48, 50, 55, 68, 71, 74, 76, 77] encompassed a range of public health initiatives, including national smoking cessation programmes [28, 50, 55, 74, 76], COVID-19 control policies [24, 71], oral health interventions [46, 48], and other measures [27, 30, 68, 77] (Table 4). A substantial increase in studies addressing COVID-19 policies was observed during 2022–2023, accounting for over half of the studies in this category [24, 28, 30, 48, 50, 55, 71].

**Table 4:** Summary of characteristics of the public health interventions (excluded screening and vaccination programmes)

No	Author, year	Population	Intervention	Complexity of interventions	Methods
1	Thavorn [74], 2008	Thai smokers	Intervention: Community pharmacist-based smoking cessation programme Comparator: Usual care	- Integrated personalised intervention combining behavioural modification and pharmacological treatment. - Pharmacists trained in counselling and smoking cessation programme design. - Linked with the national tobacco control network.	EE type: CEA Economic modelling: Combination model of decision tree and Markov models Perspective: Health system Time horizon: Lifetime Discounting: 3%*
2	Ditsuwan [27], 2013	Vehicle drivers	Interventions: Road traffic avoidance measures with 1. mass media campaigns 2. sobriety checkpoints Comparator: Do-nothing	- National-level multi-component strategies involving law enforcement, legal system, media, and public health sectors across local and national levels. - Flexible intensity of campaigns and checkpoint frequency.	EE type: CEA and CUA Economic modelling: Decision tree model Perspective: Health system Time horizon: Lifetime Discounting: 3%*
3	Tozan [77], 2014	School-aged children	Intervention: Insecticide-treated school uniforms for prevention of dengue Comparator: Do-nothing	- Implementation involved schools, families, health authorities, and manufacturing and distribution sectors.	EE type: CUA Economic modelling: Decision tree model Perspective: Societal Time horizon: 1 year Discounting: 3%*
4	Tosanguan [76], 2016	Thai smokers	Intervention: Smoking cessation programmes: counselling methods and treatments Comparator: Do-nothing	- Integrated behavioural and pharmacological interventions by trained counsellors and multidisciplinary teams. - Linked to national tobacco control. - Flexible counselling modes and treatment options.	EE type: CUA Economic modelling: Combination model of decision tree and Markov models Perspective: Societal Time horizon: Lifetime Discounting: 3%*
5	Marino [46], 2018	Primary school children in Bangkok	Intervention: Fluoridated milk programme for preventing dental caries Comparator: Usual care	- Integrated dental public health programme involving dairy companies, schools, government, public health agencies, and families.	EE type: CEA Economic modelling: Decision tree model Perspective: Societal Time horizon: 6 years Discounting: 3.25% for costs
6	Sudathip [68], 2019	Population at risk living in malaria endemic area along the Thailand borders	Intervention: Malaria elimination programmes Comparator: Do-nothing	- Implementation of integrated public health interventions with prevention, treatment, and research (e.g., surveillance, case management, capacity building, international collaboration) involved multi-level organisation (e.g., house hold, communities, local and national government organisations) across 42 endemic provinces - Integrated interventions (prevention, treatment, research, surveillance, case management, capacity building, international collaboration) across households, communities, local and national authorities. - Coverage across endemic provinces.	EE type: CBA Economic modelling: Epidemiological model Perspectives: Health system and societal Time horizon: 20 years Discounting: 3%*and 5%*
7	Nantanee [48], 2022	Children aged 9–30 months	Intervention: Fluoride varnish application programme for preventing dental caries Comparator: Usual care	- Integrated dental care (oral exam, caries risk management, hygiene instruction, varnish application) across hospitals and dental units by paediatric dentists and trained personnel, with home-based hygiene by parents.	EE type: CBA Economic modelling: Decision tree model Perspectives: Health care providers and patients Time horizon: 21 months Discounting: NA
8	Cai [24], 2023	Singaporean and Thai populations	Intervention: Bilateral COVID-19 testing and quarantine policies for bilateral travellers Comparator: Current policy	- Integrated policies across bilateral governments, ministries, tourism and aviation sectors. - Flexible testing and quarantine intervals.	EE type: CBA and CEA Economic modelling: Dynamic transmission model Perspective: Societal Time horizons: 1 month and lifetime Discounting: 3%*
9	Grant [28], 2023	Thai smokers	Intervention: National smoking cessation services Comparator: Usual care	- Integrated behavioural and pharmacological interventions by trained counsellors and multidisciplinary teams. - Linked to national tobacco control. - Flexible counselling modes and treatment options.	EE type: CUA Economic modelling: Markov model Perspective: Societal Time horizon: 50 years Discounting: 3%*
10	Janekrongtham [30], 2023	Pregnant women with HBeAg-positive and their infants.	Intervention: Antiviral prophylaxis strategies for mother-to-child transmission of HBV Comparator: Current practice	- National-level integrated strategies including maternal treatment, referral, infant vaccination and treatment, involving antenatal clinics, hospitals, labs, and health authorities. - Benefits for maternal and child health	EE type: CEA Economic modelling: Decision tree model Perspective: Health system Time horizon: 22 months Discounting: NA
11	Palakai [50], 2023	Thai smokers	Intervention: Brief Intervention service for smoking cessation in primary care Comparator: Current practice	- Integrated training workshops, online education, and community engagement across community and healthcare settings. - Required training courses for village health volunteers.	EE type: CEA Economic modelling: NA Perspective: NA Time horizon: 9 years Discounting: No discounting
12	Prasitwarachot [55], 2023	Thai smokers with COPD	Intervention: National smoking cessation services Comparator: Usual care	- Integrated behavioural and pharmacological interventions by trained counsellors and multidisciplinary teams. - Linked to national tobacco control. - Flexible counselling modes and treatment options.	EE type: CUA Economic modelling: Markov model Perspective: Societal Time horizon: lifetime Discounting: 3%*
13	Suthutvoravut [71], 2023	Asymptomatic or mild symptom COVID-19 patients	Intervention: COVID-19 isolation strategies: hospitel and community isolations Comparator: Home isolation	- Integrated quarantine across healthcare, government, and private hotel sectors with varied resources, logistics, and monitoring systems.	EE type: CEA Economic modelling: Decision tree model Perspective: Hospital Time horizon: 6 months Discounting: NA

*CBA* cost-benefit analysis, *CEA* cost-effectiveness analysis, *COPD* chronic obstructive pulmonary disease, *COVID-19* Coronavirus disease 2019, *CUA* cost-utility analysis, *EE* economic evaluation, *HBV* hepatitis B virus, *HBeAg* hepatitis B e-Antigen, *NA* not assessed, *TDF* Tenofovir Disoproxil Fumarate

*both costs and outcomes

Most evaluations primarily utilised CEA [24, 27, 30, 46, 50, 71, 74], followed by CUA [27, 28, 55, 76, 77] and CBA [24, 48, 68]. Decision tree models [27, 30, 46, 48, 71, 77] were the most common analytical approach, supplemented by hybrid models [74, 76] and Markov models [28, 55]. Studies applied either lifetime [24, 27, 55, 74, 76] or short-term horizons [30, 48, 71, 77]. The societal perspective [24, 28, 46, 55, 76, 77] was most frequently adopted, with 3% discounting applied [24, 27, 28, 55, 68, 74, 76, 77]. A minority of studies did not apply discounting [50].

#### Treatment and rehabilitation interventions

Treatment and rehabilitation interventions [29, 31–33, 51, 56, 59, 61, 67] addressed a range of medical strategies and rehabilitation services, aiming to optimise functional capacity and minimise disability (Table 5). These interventions were implemented in both hospital- [31–33, 56, 59, 61] and community care settings [29, 31, 51, 59, 61, 67], including pharmacological treatments [29, 31, 33, 51, 56, 61], lifestyle modifications [59], and integrated care approaches [33, 56, 59].

**Table 5:** Summary of characteristics of the treatment and rehabilitation interventions

No	Author, year	Population	Intervention	Complexity	Methods
1	Sritipsukho [67], 2010	Acute ischemic stroke patients and caregivers	Intervention: Home rehabilitation programme for ischemic stroke patients and caregivers Comparator: Conventional hospital care	- Integrated multidisciplinary rehabilitation across hospital and home settings.	EE type: CEA Economic modelling: EEACT Perspective: Health care providers Time horizon: 3 months Discounting: 3%*
2	Hunchangsith [29], 2012	TB patients with HIV-negative	Interventions: 1. Directly observed treatment strategies by health workers, community members, or family members 2. Mobile phone contact-reminder system Comparator: Self‑Administered Treatment	- Coordinated medication monitoring and adherence, involving families, communities, and health providers. - Flexible engagement with mobile reminders.	EE type: CUA Economic modelling: Decision tree model Perspective: Health system Time horizon: Lifetime Discounting: 3%*
3	Khiaocharoen [32], 2012	Subacute or non-acute stroke patients	Intervention: Inpatient rehabilitation services by multidisciplinary team Comparator: Usual care	- Integrated rehabilitation with multidisciplinary teams promoting self-rehabilitation behaviours in patients and caregivers.	EE type: CUA Economic modelling: EEACT Perspectives: Government and societal Time horizon: 4 months Discounting: NA
4	Saokaew [61], 2013	Patients who received warfarin therapy	Intervention: Pharmacist-participated warfarin therapy management Comparator: Usual care	- Integrated monitoring, education, communication, and safety checks by pharmacists, physicians, and healthcare payers.	EE type: CUA Economic modelling: Markov model Perspectives: Health system and societal Time horizon: Lifetime Discounting: 3%*
5	Pattanaprateep [51], 2014	Haemophilia A and B patients	Intervention: Home-based infusion of factor VIII or IX concentrates for early bleeding episodes for treating Haemophilia A and B Comparator: Current practice	- Implementation of home-based cares across patients, hospitals and healthcare payers - Requires training for patients and caregivers	EE type: CUA Economic modelling: Markov model Perspective: Government Time horizon: Lifetime Discounting: 3%*
6	Sakulsupsiri [59], 2016	Metabolic syndrome patients	Intervention: Self-management programmes at community hospital and health promoting centre Comparator: Do-nothing	- Active lifestyle and behaviour management across community and hospital settings.	EE type: CUA Economic modelling: Markov model Perspective: Societal Time horizon: Lifetime Discounting: 3%*
7	Kantito [31], 2017	Patients who received warfarin therapy	Interventions: 1. Warfarin managements with self-monitoring INR for 2. Multidisciplinary approach in anticoagulation clinic Comparator: Current practice	- Home-based self-monitoring supported and dose adjustments by training, education, telemedicine, and multidisciplinary oversight.	EE type: CUA Economic modelling: Markov model Perspectives: Health system and societal Time horizon: Lifetime Discounting: 3%*
8	Khongmee [33], 2022	HFrEF patients	Intervention: Specialised multidisciplinary care for individual patients Comparator: Current practice	- Integrated personalised services focusing on behavioural modification and education delivered by multidisciplinary teams.	EE type: CUA Economic modelling: EEACT Perspective: Societal Time horizon: 1 year Discounting: NA
9	Prayoonhong [56], 2024	T2D patients who received oral antidiabetic drugs	Intervention: Face-to-face integrated care by multidisciplinary team Comparator: Current practice	- Integrated care emphasising behavioural and lifestyle modification delivered by multidisciplinary teams.	EE type: CUA Economic modelling: EEACT Perspective: Societal Time horizon: 6 months Discounting: No discounting

*CEA* cost-effectiveness analysis, *CUA* cost-utility analysis, *EE* economic evaluation, *EEACT* economic evaluation alongside clinical trial, *HFrEF* heart failure with reduced ejection fraction, *HIV* human immunodeficiency virus, *NA* not assessed, *T2D* Type 2 diabetes, *TB* tuberculosis

*both costs and outcomes

CUA was the most frequently employed evaluating method [29, 31–33, 51, 56, 61, 67]. Analytical approaches included Markov models [31, 51, 59, 61] and EEACTs [32, 33, 56, 67]. Time horizons varied, frequently encompassing both short-term [32, 33, 56, 67] and lifetime horizons [29, 31, 51, 59, 61] to capture immediate outcomes, especially in the clinical trials [27, 28, 51, 62], and long-term outcomes. The societal perspective [31–33, 56, 59, 61] was most commonly adopted, with 3% discounting applied in the majority of studies [29, 31, 51, 59, 61, 67] to ensure consistency with the Thai health technology assessment (HTA) guidance.

### MRC definition for complex interventions

Table 6 categorises the CIs according to the five domain of MRC definition for CIs: (1) multicomponent design, (2) behavioural targeting, (3) expertise and skill requirements, (4) multitargeted group setting or level, and (5) flexibility [8]. Overall, the analysis reveals that most interventions were designed for multitargeted group setting or level [22–31, 34–47, 49–52, 54, 55, 57, 58, 62–73, 75–77, 80], with vaccination programmes exhibiting universal adherence to this domain [23, 25, 26, 35, 37, 38, 41, 42, 45, 47, 49, 58, 62, 64, 69, 70, 73, 80].

**Table 6:** MRC criteria for complex intervention

Type of intervention	MRC Domains				
	Multicomponent	Behavioural target	Expertise and skill requirement	Group/setting/level targeted	Flexibility
Screening programmes (*n* = 20)	13 (65%)	2 (10%)	8 (40%)	15 (75%)	7 (35%)
Vaccination programmes (*n* = 18)	7 (39%)	1 (6%)	0 (0%)	18 (100%)	3 (22%)
Public health intervention* (*n* = 13)	12 (92%)	1 (8%)	5 (38%)	11 (85%)	2 (15%)
Treatment and rehabilitation interventions (*n* = 9)	8 (89%)	5 (56%)	4 (44%)	4 (44%)	2 (22%)
Total (*n* = 60)	40 (67%)	9 (15%)	17 (28%)	48 (80%)	15 (25%)

*MRC* The United Kingdom Medical Research Council

*excluding screening and vaccination programmes

Multicomponent design emerged as a common feature across several interventions [22, 24, 27–34, 39, 41–48, 50, 52–56, 59–61, 64, 65, 67–72, 74, 76, 78, 79], illustrating the widespread use of integrated strategies to address health issues. This was particularly evident in public health [24, 27, 28, 30, 46, 48, 50, 55, 68, 71, 74, 76] and treatment and rehabilitation interventions [29, 31–33, 56, 59, 61, 67]. In contrast, fewer interventions required specialised expertise [28, 31, 33, 39, 40, 43, 44, 50, 53, 55, 56, 60, 67, 74, 76, 78, 81], incorporated behavioural targeting [22, 29, 32, 42, 43, 48, 56, 59, 67], or demonstrated flexibility in adapting to various contexts [22, 24, 26, 27, 29, 33, 36, 39, 41, 54, 60, 70, 79–81].

Public health interventions frequently applied multicomponent design [24, 27, 28, 30, 46, 48, 50, 55, 68, 71, 74, 76], reflecting their inherently complex health challenges and multifaceted goals. Most were directed at broadly level of targeting populations [24, 27, 28, 30, 46, 50, 55, 68, 71, 76, 77] and only one intervention incorporated behavioural components [48]. A prominent portion required specialised expertise or skills [28, 50, 55, 74, 76], though flexibility remained relatively limited [24, 27], suggesting a tendency toward structured rather than adaptive implementation.

Screening programmes showed a slightly different pattern. While flexibility was more apparent here than in other categories [22, 36, 39, 54, 60, 79, 81], behavioural components were rare [22, 43]. However, specialised expertise or skills was often required [39, 40, 43, 44, 53, 60, 78, 81], reflecting the technical competencies often associated with the screening measures. A substantial number also targeted multiple groups, settings, or levels [22, 34, 36, 39, 40, 43, 44, 53, 54, 57, 63, 65, 66, 72, 75], aligning with the selective and population-based nature of these interventions.

Vaccination programmes stood out for their uniformity in targeting multiple groups or levels, reinforcing their alignment with national-scale immunization goals. While some incorporated multiple components, behavioural elements and expertise requirements were generally absent. Flexibility was present but not a defining characteristic, indicating a focus on standardised delivery over adaptability.

Vaccination programmes displayed a complexity characteristic in targeting multiple groups, settings or levels [23, 25, 26, 35, 37, 38, 41, 42, 45, 47, 49, 58, 62, 64, 69, 70, 73, 80]. This consistency reflects their alignment with national immunisation strategies. Some interventions incorporated multiple components [41, 42, 45, 47, 64, 69, 70], flexibility attributes [41, 42, 45, 70], and behavioural targeting [42]. However, expertise and skills requirements were generally absent in this category.

Treatment and rehabilitation interventions often featured multicomponent structure (29, 31–33, 56, 59, 61, 67), whereas behavioural targeting in this category was more prevalent than another category [29, 32, 56, 59, 67]. Approximately half relied professional expertise or skills [31, 33, 56, 67], while only a few demonstrated flexibility [29, 33]. These findings underscore the specificity and complexity of treatment-focused interventions.

Most interventions met only two MRC criteria [26, 30, 32, 34, 36, 40, 45–48, 52, 53, 59, 64, 65, 68, 69, 71, 72, 74, 78–81], with few achieving four [22, 29, 39, 43, 67]. This indicates a tendency to address limited domains rather than fully meeting all criteria. The absence of any interventions meeting all five criteria underscores the importance of tailoring evaluation frameworks to the unique purposes and structural constraints of each programme type, rather than expecting uniform comprehensiveness.

### Cost components and data sources

Across the included studies, costs were classified into three principal categories: direct costs, indirect costs, and other economic impact costs, reflecting a comprehensive framework for the EE of CIs. Direct costs covered expenditures directly affecting the target population and were contingent on the nature of the interventions, for instance, vaccination expenses and administration costs [23, 25, 26, 35, 37, 38, 41, 42, 45, 47, 49, 54, 58, 64, 69, 70, 73, 80], laboratory testing costs [22, 25, 29, 30, 35, 40, 42, 44, 46, 52, 53, 56, 57, 68, 78], screening procedure costs [22, 24, 34, 36, 39, 40, 44, 52–54, 57, 60, 63, 65, 66, 72, 75, 78, 79, 81], treatment costs (e.g., for diseases or conditions [22–30, 33–37, 39–41, 44–49, 51, 52, 54–58, 63–70, 73–81], and adverse events [22, 23, 25–28, 31, 38, 41, 47, 51, 53, 55, 57–59, 61, 63, 66, 74, 77–80]), and other health care costs [27, 29, 31–33, 35, 36, 42, 45–48, 50, 56, 58–60, 62, 68, 69, 71, 72, 74–77, 80, 81]. Additionally, expenses related to transportation [23, 25, 29, 31–36, 38–40, 44–47, 49, 52, 53, 55–58, 60–63, 65, 66, 72, 76–78, 81], food [23, 31, 32, 34, 36, 38, 40, 44, 45, 47, 49, 52, 53, 55–58, 60, 61, 63, 65, 66, 77–81], accommodation [38, 40, 44, 49, 53, 56, 58, 65, 79–81] incurred during the intervention were incorporated into this category.

Indirect costs primarily relate to productivity losses attributable to illness or caregiving responsibilities, such as forgone income by patients or caregivers [23, 25, 29, 32, 33, 35, 39, 40, 44–47, 58, 60, 62, 65, 66, 68, 75, 76, 81], and other costs related to premature morbidity and mortality [24, 27, 40, 44, 46, 54, 56, 57, 68, 78–81]. Notably, certain studies, especially CUAs, excluded indirect costs to mitigate the risk of double-counting health utility losses attributable to illness, adhering to the Thai HTA guidance [26, 28, 36, 37, 48–53, 55, 57, 61, 63, 65].

Other economic impact costs addressed condition-specific consequences, including the health burdens linked to malaria [68] or COVID-19 [24]. These cost classifications underscore the multifaceted economic consequences associated with healthcare decisions and public health interventions, such as the economic growth losses incurred due to COVID-19 quarantine measures [24]. Several vaccination programmes referred to externality costs, such as the benefits of herd immunity, which extends protection to unvaccinated populations by infectious disease transmission [26, 35–37, 41, 42, 49, 54, 58, 69, 73]. However, none of the studies formally accounted for these costs in their analysis.

Cost data were obtained from diverse array of sources, including direct survey [28, 32, 33, 35, 39, 44, 48, 49, 54, 56–58, 65, 75, 78], published literature [23–31, 34, 37–42, 45, 47, 49, 52–56, 58, 59, 61–65, 69, 74–80], national [25, 26, 35, 37, 42, 45, 49, 51, 53, 66, 68–70, 78, 80] or hospital databases [22, 27, 35, 36, 44, 50, 56, 57, 60, 65, 71, 73–75], Thailand ‘s standard cost list [23, 36, 55, 60, 76], cohort studies [40], clinical trials [43, 46, 59, 67, 72, 81] and other sources [36, 58, 64, 69, 71, 74, 76, 80]. The majority of included studies collected cost data in accordance with the study perspective. Nevertheless, certain studies that declared the societal perspective only accounted for intervention costs while neglecting broader costs [41, 59]. Furthermore, Leelukkanaveera et al. [43] did not provide detailed cost information, limiting the comprehensiveness of their analysis.

### Outcome measures and data sources

The health outcomes assessed across the included studies were diverse and aligned with the specific type of EE undertaken. CUAs frequently employed QALYs [23, 26, 28, 31–40, 42–45, 49, 51–63, 65, 66, 73, 75, 76, 78, 81] and DALYs [25, 27, 29, 41, 47, 69, 77] to quantify improvements in health and reductions in disease burden. In contrast, CEAs prioritised a range of disease-specific or clinical effectiveness indicators, including cases [22–24, 30, 41, 64, 70, 72, 77] or deaths averted [23, 38, 50, 64], life-time gained [38, 43, 49, 50, 60, 62, 74, 75, 80], and measures related to functional or disability outcomes [27, 32, 47, 48, 50, 64, 67, 71]. Meanwhile, cost-benefit analyses expressed outcomes in monetary terms, incorporating net economic gains [24] and willingness-to-pay for improved health outcomes [24, 48, 68, 79]. This heterogeneity in outcome measures illustrates the complexity and multidimensionality of the interventions under evaluation, aiming to comprehensive may capture both their clinical efficacy and economic implications. Outcome data were obtained from variety sources, including published literature, government reports, clinical trials and survey [22–81].

### Identified limitations

The limitations identified across the included studies centred on three key areas: data quality, methodological assumptions, and generalisability. Numerous studies encountered challenges associated with underestimated [23, 27, 38, 66, 73, 74], incomplete [22–24, 26, 29, 30, 33, 58, 60], outdated data [22, 24, 26, 38], or data absence, particularly in resource-constrained settings where reliable information was often scarce [22, 28–30, 33–36, 38, 39, 41, 45, 49, 50, 53–55, 57, 58, 61, 63, 65, 66, 68, 69, 73–78, 80]. Methodological limitations frequently arose from reliance on models and assumptions that did not fully capture the complexities of real-world scenarios [22, 23, 25–27, 30, 31, 33, 36–38, 40, 41, 44, 48, 49, 52, 54, 56, 59, 60, 64, 65, 69, 70, 75, 76, 79]. These included degree of severity [23, 33, 35, 53, 58], indirect effects [27, 34, 36, 41, 42, 48, 50, 51, 53, 54, 67, 71], behavioural factors [24, 42, 59, 64, 76], long-term impacts [22, 26, 30, 42, 56, 64, 67, 71, 76], non-health benefits [24, 37, 42, 46, 71, 76], and externality such as herd immunity [26, 35–37, 41, 42, 49, 54, 58, 69, 73]. Furthermore, dependence on assumptions, such as those related to disease incidence or prevalence [22–24, 35, 38, 40, 41, 47, 53, 75], feasibility [30, 39, 42, 43, 48, 52–54, 60, 63, 64, 79], accessibility [22, 25, 30, 39, 48, 52, 54, 63, 79], effectiveness of the interventions [23, 27, 29, 31, 34, 36, 39, 47, 64, 66, 67, 80], and intervention or administration costs [25, 27, 30, 35, 37, 38, 44, 47, 58, 60, 63, 65, 67], often reduced the robustness of the findings. The generalisability of results was frequently constrained by the specificity of individual studies to particular regions, populations, or economic contexts, thereby limiting the applicability of conclusions on a broader scale [22–28, 30, 31, 33, 35, 36, 38, 40, 41, 44, 47, 48, 52, 53, 55–61, 65, 67, 70, 76, 78–80].

### Assessment of methodological consistency with the MRC framework

To contextualise Thailand’s current practices in EE of CIs, an evaluation of economic consideration of the MRC framework with findings from the Thai studies was compared. The MRC guidance [6] emphasises the importance of integrating EE from the developing and feasibility processes of CI development. This includes cost assessments, pilot modelling, and value of information analyses to inform feasibility and design decisions. Whereas, most of included EEs in Thailand typically estimated future economic consequences by using the economic modelling techniques [22–31, 34–55, 57–66, 68–71, 73–81]. The post hoc approach to reduces the potential uncertainty of data were used in the EEACTs [32, 33, 56, 67, 72]. Furthermore, the limited use of value of information analyses in a few study [42, 80]. This highlights a missed opportunity to strengthen intervention design through economic insights.

#### Methodological approaches

The MRC guidance promotes flexible EE methods, particularly CCA and CBA, as they accommodate the multidimensional outcomes of CIs [6]. Conversely, the Thai evaluations showed a heavy reliance on CUA [23, 25–29, 31–45, 47, 49, 51–63, 65, 66, 69, 73, 75–78, 81] along with the Thai HTA guidance [82]. While CUA facilitates comparability across interventions by quantifying outcomes in QALYs or DALYs, it may fail to capture non-health benefits and nuanced social outcomes. The underuse of CCA and CBA suggests a methodological rigidity in the Thai context, which may limit the evaluative scope for more complex or intersectoral interventions. This associated with the types of intervention that CBA resented in public health interventions [24, 48, 68] and screening programme [79].

However, a few studies attempted to analyse with more than one EE framework by combining CEA with CUA to examine diverse consequences [24, 25, 27]. The limited use of these broader methods in Thailand may constrain the evaluative capacity needed for more nuanced or cross-sectoral interventions.

#### Perspective

The MRC guidance highlights the importance of considering a broad range of perspectives, however; it does not mandate the societal perspective as the default approach [6]. In Thailand, the national guidance recommend adopting the societal perspective as the primary analysis, supplemented by other perspectives where feasible [82]. While the majority of the studies [24, 26, 28, 31–41, 43–47, 49, 52–63, 65, 66, 68, 75–81] claimed to use a societal perspective. Some of them were narrow in practice, frequently omitting important components such as productivity loss, caregiver burden, and broader social impacts [26, 41, 59]. In some cases, multiple perspectives were analysed to present the economic costs and consequences in different standpoints, particularly in consideration of health budget constrain [31, 32, 37, 43, 47, 48, 54, 58, 60, 61, 65, 68, 79, 81].

#### Modelling techniques

Economic models are critical in assessing the EE of CIs, especially long-term impacts. The selection of an appropriate economic model is contingent upon the complexity of the intervention. The MRC recommends sophisticated modelling techniques such as dynamic transmission models, microsimulations, or system dynamics to reflect the nonlinear and interactive nature of CIs [6]. In Thailand, hybrid models combining decision trees and Markov processes were widely used, particularly in screening [34, 36, 44, 52, 53, 57, 60, 63, 65, 75, 78] and vaccination programmes [23, 26, 37, 38, 41, 42]. Advanced models capable of simulating feedback loops, adaptive behaviours, or pathway dynamics were rarely used. This represents a technical limitation that restricts the evaluation of more dynamic or policy-driven interventions. However, dynamic transmission models were primarily used in the vaccination programmes [45, 69, 70, 80] to account for dynamic pathway of infectious diseases and herd immunity effects. Furthermore, public health interventions also concerned the advance modelling technique, for instance, dynamic transmission model for bilateral national COVID-19 control policy [24] and epidemiological model for malaria prevention programmes [68]. However, the treatment and rehabilitation intervention preferred EEACTs over economic modelling [32, 33, 56, 67]. As they provided a structured framework for evaluating costs and outcomes to assess with-in clinical benefits.

Furthermore, expanding the scope of economic models to incorporate societal and externality effects was major concerns in several studies, as this would provide a more comprehensive perspective on the impacts of interventions [26, 35, 37, 38, 40, 42, 45, 58, 68, 74]. Scenario-based analysis was suggested to assess the robustness of conclusions across varying assumptions and contexts [30, 31, 39, 42, 69], thereby creating models that better reflected the complexities of real-world conditions across diverse contexts in Thailand [31, 37, 42, 45, 53, 67, 71, 77, 79].

#### Time horizon

Long-term horizons (≥ 20 years or lifetime) were predominantly utilised across all categories [24, 26–29, 31, 34, 36–44, 49, 51–55, 57, 59–61, 63, 65, 66, 68, 73–76, 78, 79, 81]. This pattern appears consistent with the MRC framework, which highlights the importance of considering long-term evaluation [6]. These horizons reflected their long-term impacts on disease prevention, mortality reduction, and healthcare resource allocation and quality of life. In contrast, short-term horizons (≤ 1 year) were significantly employed in treatment and rehabilitation interventions [32, 33, 56, 67], reflecting the emphasis on immediate clinical outcomes. Medium-term horizons (up to 10 years) were applied to the public health interventions [30, 46, 48, 50] to capture intermediate effects without requiring lifetime consequences. This pattern aligns with Thai HTA guidelines [82], which recommend selecting a time horizon long enough to reflect the full effects of an intervention, while also underscoring the importance of matching horizon choice to the nature and sustainability of the intervention’s impacts.

Economic models that incorporate long-term health outcomes, such as Markov and hybrid models, were particularly relevant for the interventions with long-term benefits [26, 31, 34, 36–39, 41–44, 49, 51–55, 57, 59–61, 63, 65, 66, 73–76, 78], such as the vaccinations and screening programmes. Conversely, decision tree model and short-term analyses seem to be more preferable for interventions with immediate outcomes, such as clinical interventions [22, 35, 71, 77]. By employing suitable economic modelling techniques and time horizons, policymakers should optimise resource allocation, ensuring that CIs achieve maximal benefits.

#### Stakeholder engagement

The MRC highlights the role of stakeholder engagement throughout the evaluation process to ensure contextual relevance and policy applicability [6]. Stakeholder involvement in Thailand contrasts sharply with practices in high-income countries, where engagement is a cornerstone of EEs. For instance, in the United Kingdom, stakeholder involvement is routinely integrated into HTA processes to enhance the relevance and acceptance of findings [83].In the Thai context, stakeholder involvement was infrequently reported and it is unclear that they had integrated into the evaluation methodology. This disconnect may reduce the uptake of economic evidence in decision-making processes, particularly for CIs requiring intersectoral collaboration. However, the stakeholder engagement might be done in another process of the CI implementation but did not mention on the EE report.

#### Implementation, generalisability, and real-world context

The MRC framework encourages researchers to incorporate considerations of consider scalability, feasibility, and contextual adaptability into evaluation planning where appropriate [6]. While some Thai studies included real-world implementation considerations, such efforts were inconsistent, particularly in resource-constrained settings where reliable information was often scarce [22, 28–30, 33–36, 38, 39, 41, 45, 49, 50, 53–55, 57, 58, 61, 63, 65, 66, 68, 69, 73–78, 80]. The utilisation of updated and high-quality data sources was considered essential for enhancing the accuracy and reliability of findings [22, 23, 26, 36–38, 79]. Evaluations of CIs were encouraged to prioritise inclusivity in country-specific data collection, encompassing diverse geographic regions within Thailand to improve the applicability and relevance of results [22, 23, 25–27, 38, 52, 55, 68]. It was also recommended that longitudinal studies be employed to capture the full spectrum of effects in real-world contexts, including long-term outcomes and lifetime impacts [22, 48, 55, 56, 67, 68, 77]. The generalisability of results was frequently constrained by the specificity of individual studies to particular regions, populations, or economic contexts, thereby limiting the applicability of conclusions on a broader scale [22–28, 30, 31, 33, 35, 36, 38, 40, 41, 44, 47, 48, 52, 53, 55–61, 65, 67, 70, 76, 78–80]. Therefore, in Thailand, the development of adaptable frameworks was proposed to facilitate the application of findings across diverse contexts, thereby enhancing generalisability [22, 39, 42, 44, 45, 53, 68, 71, 74]. Collectively, these recommendations aimed to refine methodologies and ensure that future research generated more actionable and universally applicable insights.

### Quality assessment

**Fig. 2:**
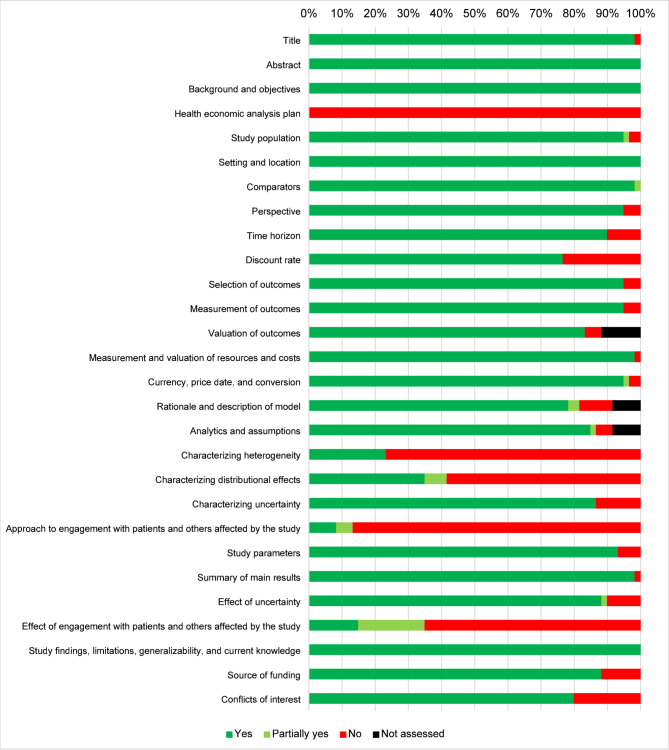
Quality assessment of the included studies

The quality assessment of the includes articles are detailed in Supplement 4 and the quality assessment for each item across all included studies are detailed in Fig. 2. Most studies have fulfilled 23 items, for instance, abstract, background and objectives, setting and location, and cost measurement. However, stakeholders’ engagement, together with reporting of the effect on different groups of stakeholders, characterising heterogeneity and characterising distributional effects were the major methodological challenges across the included studies. Moreover, five non-model-based studies were not applicable for items related to modelling issues [32, 33, 56, 67, 72].

## Discussion

This systematic review provides a comprehensive synthesis of the economic considerations and methodological approaches employed in the EE of CIs in Thailand. Drawing from 60 included studies [22–81], the findings provide an essential overview of the evaluation of EE practice over the past decades, addressing various intervention types, methodological frameworks, and the challenges arising from increased policy interest in assessing the value of CIs. Approximately 25% of included studies were published in recent years [23, 24, 28, 30, 36, 40, 44, 49, 50, 55, 56, 64, 65, 71], reflecting increased research activities. This growth may be attributed to advancements in healthcare technology and an evolving healthcare landscape, particularly in response to global health crises such as the COVID-19 pandemic. These developments have facilitated a more comprehensive assessment of the long-term benefits of interventions. While the adoption and application of EE in evaluating CIs have expanded under diverse contextual constraints, several methodological challenges persist that limited full alignment with MRC guidance for CIs in the Thai context.

An analysis of the included interventions reveals considerable variability in how they align with the five MRC domains: multicomponent design, behavioural targeting, expertise and skill requirements, multitargeted group or setting, and flexibility. Several interventions demonstrated a broad and integrative approach, often combining multiple components and addressing various target groups or settings. However, flexibility and behavioural targeting were less frequently emphasised, suggesting limited adaptability and personalisation in their design. Vaccination programmes consistently targeted diverse groups and settings, reflecting their large-scale public health objective. In contrast, treatment and rehabilitation interventions placed greater emphasis on behavioural targeting and individualised designs. A potential limitation arises from the inclusion criteria, whereby interventions meeting at least one inclusion criterion may risk over-classification. However, this approach was deemed appropriate to ensure that the included interventions with a single qualifying feature but complicated development or implementation processes were not excluded, thereby capturing the full breadth of intervention characteristics described by the MRC and accommodating diverse perspectives on complexity.

Despite incorporating characteristics associated with complexity, none of the interventions explicitly identified themselves as complex interventions. This absence of self-identification indicates a conceptual gap. Promoting early integration of CI concept into EE in Thailand could strengthen the use of economic evidence in designing and optimising CIs to ensure that complexity is systematically addressed throughout the evaluation process.

A strong preference for CUA was observed, driven primarily by adherence to the national guidelines. While CUA offer standardisation metrics for comparison overall health improvements, they may inadequately reflect the broader, often non-health-benefits of CIs. Alternative approaches such as CBA and CCA, which are more suited for capturing multidimensional impacts, were underutilised. CBA extends beyond direct health benefits by incorporating economic and wider social consequences in terms of monetary values. This indicates methodological rigidity that could constrain comprehensive policy assessment for cross-sectoral interventions. The multidimensional nature of these approaches may result in greater variability in findings, necessitating a more comprehensive assessment of an intervention’s broader implications. The predominance of model-based EEs, especially a combination of decision tree and Markov models, reflects to capture the complexity nature of CIs. However, the limited application of more advanced techniques, such as dynamic transmission or system dynamics models, suggests a technical gap in modelling the non-linear and adaptive behaviours typical in CIs. While most studies adopted a long-term horizon, which is appropriate for capturing ultimate intervention impacts and the optimisation of health resources and social well-being. Short-term timeframes remained common in EEACTs, potentially underestimating the long-term value of interventions.

Additionally, challenges related to data variability and limited access to reliable local data are consistent with findings from other low- and middle-income countries. The lack of robust epidemiological and cost data in resource-constrained settings contrasts with well-established EE countries, where more comprehensive health data systems enable greater evaluation precision and result generalisability [84, 85].

### Strengths and limitations

This review underscores the need for a Thailand-specific evaluation framework that address the unique challenges of assessing CIs. By applying both the CHEERS checklist and the MRC framework, this study provides a nuanced understanding of the quality, design, and methodological alignment of EEs in Thailand. Its broad scope, that covers diverse intervention types and multiple EE methods, offers valuable insights into evolving trends within Thailand ‘s EE landscape.

However, this study has several limitations. First, study selection and data extraction were conducted by a single reviewer, with subsequent validation by a second reviewer demonstrating substantial agreement (Cohen’s kappa = 0.81). However key decisions regarding study eligibility were discussed collaboratively with the research team. Second, the exclusion of non-English-language publications introduces potential language bias, although a comprehensive search strategy was employed to ensure broad coverage of the literature. The 60 included articles represent a reasonable sample for characterising EE practices related to CIs in Thailand. Another limitation concerns the lack of a formal national definition of CIs. This review adopted the 2023 MRC definition [6], which, although widely accepted (89, 90), may have excluded interventions aligned with alternative definitions (86–88), thus narrowing the scope of the study’s scope.

Although Thai EEs partially align with the MRC framework, significant gaps remain. Thailand has made important strides in adopting structured EE methods, particularly through model-based approaches and the use of societal perspectives, significant gaps remain in aligning with the MRC framework. These include limited early economic integration, overreliance on narrow evaluation methods, minimal stakeholder engagement, and underutilisation of advanced modelling techniques. Addressing these shortcomings would enhance the methodological rigor, policy relevance, and real-world impact of EEs in Thailand. By adopting more flexible and participatory approaches, Thailand can strengthen its capacity to evaluate and implement CIs that improve both health outcomes and resource efficiency.

Future research should adopt a holistic perspective that captures the full spectrum of CI benefits, rather than assessing isolated components. This requires using not only CUA but also CCA and CBA to evaluating cross-sectoral impacts. Advanced modelling approaches should be applied to reflect real-world complexity and non-linear effects. Integrating stakeholder engagement into the design and implementation of EEs is essential to enhance relevance, transparency, and policy applicability. Strengthening data infrastructure, through the development of region-specific databases and the promotion of collaborative data sharing, will improve modelling accuracy, reduce reliance on assumptions, and increase the credibility of findings.

## Conclusion

The review provides the comprehensive examination of methodological approaches in the EE of CIs in Thailand since 2008. It highlights both advancements and ongoing challenges in aligning the Thai practices with the MRC guidance. Current practices demonstrated diverse methodological approaches and partial alignment with the MRC framework. However, further improvement is needed to effectively evaluate highly intricate, multifaceted interventions that produce multidimensional impacts. Strengthening evaluation requires adopting a societal perspective that captures cross-sectoral impacts, enhancing stakeholder engagement, and developing more comprehensive data systems. This framework would support the generation of robust, policy-relevant evidence to guide the design, prioritisation, and implementation of CIs within the Thai health system.

## Supplementary Information

Supplementary Material 1.

## Data Availability

All data supporting the findings of this study are available within the paper and its Supplementary Information.

## Abbreviations

CBA: Cost-benefit analysis
CCA: Cost-consequence analysis
CEA: Cost-effectiveness analysis
CHEERS: Consolidated Health Economic Evaluation Reporting Standards statement
CIs: Complex interventions
CUA: Cost-utility analysis
DALYs: Disability-adjusted life-years
EE: Economic evaluation
EEACTs: Economic evaluation alongside clinical trials
HTA: Health technology assessment
MRC: United Kingdom Medical Research Council
PRISMA: Preferred Reporting Items for Systematic Reviews and Meta-Analyses
PROSPERO: Prospective Register of Systematic Reviews
QALYs: Quality-adjusted life-years
RCTs: Randomised controlled trials
