# Supplementary material for: Approaches to economic evaluations of complex interventions in thailand: a systematic review: Author
Source: BMC Public Health. 2025 Nov 25;25:4135. doi: 10.1186/s12889-025-25486-y (PMC12648963; doi:10.1186/s12889-025-25486-y)
Supplement: Supplementary file 1 — Supplementary Material 1. [file 12889_2025_25486_MOESM1_ESM.docx]

**Additional file 1**

Contents

[Supplement table 1. PICOS criteria 2](#_Toc212449385)

[Supplement table 2. Search Terms 3](#_Toc212449386)

[Databases: Medline and Embase (Ovid) 3](#_Toc212449387)

[Database: EconLit (EBSCO) 5](#_Toc212449388)

[Supplement table 3. Eligibility criteria 6](#_Toc212449389)

[Supplement table 4. Summary of quality assessment score of the included studies 7](#_Toc212449390)

[References 10](#_Toc212449391)

# **Supplement table 1. PICOS criteria**

| **Domains** | **Details** |
| --- | --- |
| **Population** | 1. Thai residents  2. Populations affected by Thai health policies or Thai health interventions (e.g., immigrants, and travellers) |
| **Intervention** | Any complex intervention in health |
| **Comparator** | Any relevant comparator |
| **Outcome** | Any kind of economic evaluation outcomes (e.g., incremental-cost effectiveness ratio, net monetary benefit, and return of investment) |
| **Setting** | Economic evaluation studies (i.e., cost-benefit analysis, cost-consequence analysis, cost-effectiveness analysis, cost-maximisation analysis, and cost-utility analysis) |

# **Supplement table 2. Search Terms**

## Databases: Medline and Embase (Ovid)

| # | Search Term |
| --- | --- |
| 1 | Economics/ |
| 2 | "costs and cost analysis"/ |
| 3 | Cost allocation/ |
| 4 | Cost-benefit analysis/ |
| 5 | Cost control/ |
| 6 | Cost savings/ |
| 7 | Cost of illness/ |
| 8 | Cost sharing/ |
| 9 | "deductibles and coinsurance"/ |
| 10 | Medical savings accounts/ |
| 11 | Health care costs/ |
| 12 | Direct service costs/ |
| 13 | Drug costs/ |
| 14 | Employer health costs/ |
| 15 | Hospital costs/ |
| 16 | Health expenditures/ |
| 17 | Capital expenditures/ |
| 18 | Value of life/ |
| 19 | Exp economics, hospital/ |
| 20 | Exp economics, medical/ |
| 21 | Economics, nursing/ |
| 22 | Economics, pharmaceutical/ |
| 23 | Exp "fees and charges"/ |
| 24 | Exp budgets/ |
| 25 | (low adj cost).mp. |
| 26 | (high adj cost).mp. |
| 27 | (health?care adj cost$).mp. |
| 28 | (fiscal or funding or financial or finance).tw. |
| 29 | (cost adj estimate$).mp. |
| 30 | (cost adj variable).mp. |
| 31 | (unit adj cost$).mp. |
| 32 | (economic$ or pharmacoeconomic$ or price$ or pricing).tw. |
| 33 | OR/1-32 |
| 34 | intervent |
| 35 | health plan implementation.mp. |
| 36 | Reimbursement, Incentive.mp. |
| 37 | Quality Improvement.mp |
| 38 | exp quality control/ |
| 39 | Patient Care Management.mp. |
| 40 | exp. Health service/ |
| 41 | exp. mass screening/ |
| 42 | Prospective payment assessment commission.mp. |
| 43 | Pay for performance pr incentive* or screening or vaccine or immuni?ation or quality or knowledge transfer or implement*).mp or intervention/.ti,ab. |
| 44 | exp public health/ |
| 45 | ((intervention$ or component$ or parameter$) adj2 (multiple or complex or multi faceted or multifaceted or multi parameter or multiparameter or multi component or multicomponent)).ti,ab. |
| 46 | OR/ 34-45 |
| 47 | Exp thai/ |
| 48 | Thai/. ti,ab. |
| 49 | OR /47-49 |
| 50 | #33 AND #46 AND #49 |

## Database: EconLit (EBSCO)

| # | Search Terms |
| --- | --- |
| 1 | Socioeconomics |
| 2 | Cost benefit analysis |
| 3 | Cost effectiveness analysis |
| 4 | Cost of illness |
| 5 | Cost control |
| 6 | Economic aspect |
| 7 | Financial management |
| 8 | Health care cost |
| 9 | Health care financing |
| 10 | Health economics |
| 11 | Hospital cost |
| 12 | TI(fiscal or financial or finance or funding). |
| 13 | Cost minimization analysis |
| 14 | cost estimate |
| 15 | cost variable |
| 16 | unit cost* |
| 17 | #1 OR #2 OR… OR #16 |
| 18 | intervention* or component* or parameter* |
| 19 | multiple or complex or multi faceted or multifaceted or multi parameter or multiparameter or multi component or multicomponent |
| 20 | #18 AND #19 |
| 21 | thai* |
| 22 | #17 AND #19 AND # 21 |

# **Supplement table 3. Eligibility criteria**

| **PICOS** | **Inclusion criteria** | **Exclusion criteria** |
| --- | --- | --- |
| **P** | 1. Thai residents  2. Populations who affected from Thai health policies or health interventions (e.g., immigrants, travellers, expatriates and other relevant stakeholders) | None |
| **I** | Any kinds of complex interventions according to the MRC criteria:  1. Multicomponent design  2. Behavioural targeting  3. Expertise and skill requirements  4. multi-targeted group setting or level  5. Flexibility | None |
| **C** | Omitted | None |
| **O** | Omitted | None |
| **S** | Economic evaluation studies (i.e., cost-benefit analysis, cost-consequence analysis, cost-effectiveness analysis, cost-maximisation analysis, and cost-utility analysis) | Cost comparation  Cost description |
| **Others** | Published from 2008 until July 2024 | Non-human studies  Non-peer-review articles  Narrative and systematic reviews  Non-English language articles |

# **Supplement table 4.** **Summary of quality assessment score of the included studies**

| No | Author, year | Title | Abstract | Background and objectives | Health economic analysis plan | Study population | Setting and location | Comparators | Perspective | Time horizon | Discount rate | Selection of outcomes | Measurement of outcomes | Valuation of outcomes | Measurement and valuation of resources and costs | Currency, price date, and conversion | Rationale and description of model | Analytics and assumptions | Characterizing heterogeneity | Characterizing distributional effects | Characterizing uncertainty | Approach to engagement with patients and others affected by the study | Study parameters | Summary of main results | Effect of uncertainty | Effect of engagement with patients and others affected by the study | Study findings, limitations, generalizability, and current knowledge | Source of funding | Conflicts of interest |
| --- | --- | --- | --- | --- | --- | --- | --- | --- | --- | --- | --- | --- | --- | --- | --- | --- | --- | --- | --- | --- | --- | --- | --- | --- | --- | --- | --- | --- | --- |
|  |  | 1 | 2 | 3 | 4 | 5 | 6 | 7 | 8 | 9 | 10 | 11 | 12 | 13 | 14 | 15 | 16 | 17 | 18 | 19 | 20 | 21 | 22 | 23 | 24 | 25 | 26 | 27 | 28 |
| 1 | Thavorn et al.(1), 2008 | Y | Y | Y | N | Y | Y | Y | Y | Y | Y | Y | Y | NA | Y | Y | Y | Y | N | N | Y | N | Y | Y | Y | N | Y | Y | Y |
| 2 | Tengtrisorn et al.(2), 2009 | Y | Y | Y | N | Y | Y | Y | N | N | N | Y | Y | NA | Y | Y | NA | NA | N | Y | Y | Y | Y | N | Y | Y | Y | N | N |
| 3 | Chotivitayatarakorn et al.(3), 2010 | Y | Y | Y | N | Y | Y | Y | Y | N | Y | Y | Y | Y | Y | Y | Y | Y | N | Y | Y | N | Y | Y | Y | N | Y | N | N |
| 4 | Leelukkanaveera et al.(4), 2010 | Y | Y | Y | N | Y | Y | Y | Y | Y | Y | N | N | N | N | Y | N | N | N | N | N | N | N | Y | Y | Y | Y | Y | Y |
| 5 | Sritipsukho et al.(5), 2010 | Y | Y | Y | N | Y | Y | Y | Y | Y | Y | Y | Y | Y | Y | Y | NA | NA | N | Y | Y | N | Y | Y | Y | Y | Y | Y | N |
| 6 | Lee et al.(6), 2011 | Y | Y | Y | N | Y | Y | Y | Y | N | Y | Y | Y | Y | Y | Y | Y | Y | N | Y | Y | N | Y | Y | Y | Y | Y | Y | N |
| 7 | Leelahavarong et al.(7), 2011 | Y | Y | Y | N | Y | Y | Y | Y | Y | Y | Y | Y | Y | Y | Y | Y | Y | Y | Y | Y | N | Y | Y | Y | Y | Y | Y | Y |
| 8 | Praditsitthikorn et al.(8), 2011 | Y | Y | Y | N | Y | Y | Y | Y | Y | Y | Y | Y | Y | Y | Y | Y | Y | N | Y | Y | N | Y | Y | Y | Y | Y | Y | Y |
| 9 | Hunchangsith et al.(9), 2012 | Y | Y | Y | N | Y | Y | Y | Y | Y | Y | Y | Y | Y | Y | Y | Y | Y | N | N | Y | N | Y | Y | Y | N | Y | Y | Y |
| 10 | Khiaocharoen et al. (10), 2012 | Y | Y | Y | N | Y | Y | Y | Y | Y | N | Y | Y | Y | Y | N | NA | NA | N | N | Y | N | Y | Y | Y | N | Y | Y | Y |
| 11 | Kingkaew et al. (11), 2012 | Y | Y | Y | N | Y | Y | Y | Y | Y | Y | Y | N | Y | Y | Y | Y | Y | N | Y | Y | N | Y | Y | Y | Y | Y | Y | Y |
| 12 | Muangchana et al.(12), 2012 | Y | Y | Y | N | Y | Y | Y | Y | Y | Y | Y | Y | Y | Y | Y | Y | Y | N | Y | Y | N | Y | Y | Y | Y | Y | N | N |
| 13 | Termrungruanglert et al.(13), 2012 | Y | Y | Y | N | Y | Y | Y | Y | Y | Y | Y | Y | Y | Y | Y | Y | Y | N | N | Y | N | Y | Y | Y | N | Y | N | Y |
| 14 | Ditsuwan et al.(14), 2013 | Y | Y | Y | N | N | Y | Y | Y | Y | Y | Y | Y | Y | Y | Y | N | Y | N | Y | Y | N | Y | Y | Y | N | Y | Y | Y |
| 15 | Kulpeng et al.(15), 2013 | Y | Y | Y | N | N | Y | Y | Y | Y | Y | Y | Y | Y | Y | Y | Y | Y | N | N | Y | N | Y | Y | Y | N | Y | Y | N |
| 16 | Rattanavipapong et al.(16), 2013 | Y | Y | Y | N | Y | Y | Y | Y | Y | Y | Y | Y | Y | Y | Y | Y | Y | N | N | Y | N | Y | Y | Y | Y | Y | Y | Y |
| 17 | Saokaew et al.(17), 2013 | Y | Y | Y | N | Y | Y | Y | Y | Y | Y | Y | Y | Y | Y | Y | Y | Y | N | N | Y | N | Y | Y | Y | Y | Y | Y | Y |
| 18 | Pattanaprateep et al.(18), 2014 | Y | Y | Y | N | Y | Y | Y | Y | Y | Y | Y | Y | Y | Y | Y | Y | Y | N | N | Y | N | Y | Y | Y | N | Y | Y | Y |
| 19 | Sangmala et al. (19), 2014 | Y | Y | Y | N | Y | Y | Y | Y | Y | Y | Y | Y | Y | Y | Y | Y | Y | N | Y | Y | N | Y | Y | Y | Y | Y | Y | N |
| 20 | Saokaew et al.(20), 2014 | Y | Y | Y | N | Y | Y | Y | Y | Y | Y | Y | Y | Y | Y | Y | Y | Y | Y | N | Y | N | Y | Y | Y | N | Y | Y | N |
| 21 | Srisubat et al.(21), 2014 | Y | Y | Y | N | Y | Y | Y | Y | Y | Y | Y | Y | Y | Y | N | Y | Y | Y | N | Y | N | Y | Y | Y | Y | Y | Y | Y |
| 22 | Tozan et al. (22), 2015 | Y | Y | Y | N | Y | Y | Y | Y | Y | Y | Y | Y | Y | Y | Y | N | Y | N | Y | Y | N | Y | Y | Y | Y | Y | Y | Y |
| 23 | Thiboonboon et al.(23), 2015 | Y | Y | Y | N | Y | Y | Y | Y | Y | Y | Y | Y | Y | Y | Y | Y | Y | N | N | Y | N | Y | Y | Y | N | Y | Y | Y |
| 24 | Sakulsupsiri et al.(24), 2016 | Y | Y | Y | N | Y | Y | Y | Y | Y | Y | Y | Y | Y | Y | Y | Y | Y | Y | N | Y | N | Y | Y | Y | Y | Y | Y | Y |
| 25 | Tosanguan et al.(25), 2016 | Y | Y | Y | N | Y | Y | Y | Y | Y | Y | Y | Y | Y | Y | Y | Y | Y | N | N | Y | N | Y | Y | Y | N | Y | Y | Y |
| 26 | Kantito et al.(26), 2017 | Y | Y | Y | N | Y | Y | Y | Y | Y | Y | Y | Y | Y | Y | Y | Y | Y | N | Y | Y | N | Y | Y | Y | N | Y | N | Y |
| 27 | Kittikraisak et al.(27), 2017 | Y | Y | Y | N | Y | Y | Y | Y | Y | Y | Y | Y | Y | Y | Y | Y | Y | N | Y | Y | N | Y | Y | Y | Y | Y | Y | Y |
| 28 | Kotirum et al.(28), 2017 | Y | Y | Y | N | Y | Y | Y | Y | Y | Y | Y | Y | Y | Y | Y | Y | Y | N | Y | Y | N | Y | Y | Y | Y | Y | Y | Y |
| 29 | Phisalprapa et al.(29), 2017 | Y | Y | Y | N | Y | Y | Y | Y | Y | Y | Y | Y | Y | Y | Y | Y | Y | N | N | Y | N | Y | Y | Y | N | Y | N | N |
| 30 | Wongwai et al.(30), 2017 | Y | Y | Y | N | Y | Y | Y | Y | Y | Y | Y | Y | Y | Y | Y | Y | Y | N | N | Y | N | Y | Y | Y | N | Y | Y | Y |
| 31 | Kumdee et al.(31), 2018 | Y | Y | Y | N | Y | Y | Y | Y | Y | Y | Y | Y | Y | Y | Y | Y | Y | N | N | Y | Y | Y | Y | Y | N | Y | Y | Y |
| 32 | Marino et al.(32), 2018 | Y | Y | Y | N | Y | Y | Y | Y | Y | Y | Y | Y | Y | Y | Y | N | N | N | N | Y | N | N | Y | Y | N | Y | Y | Y |
| 33 | Dilokthornsakul et al.(33), 2019 | Y | Y | Y | N | Y | Y | Y | Y | Y | Y | Y | N | Y | Y | Y | Y | Y | N | Y | Y | Y | Y | Y | Y | Y | Y | N | N |
| 34 | Phisalprapa et al.(34), 2019 | Y | Y | Y | N | Y | Y | Y | Y | Y | Y | Y | Y | Y | Y | Y | Y | Y | N | N | Y | N | Y | Y | Y | N | Y | Y | Y |
| 35 | Saokaew et al.(35), 2019 | Y | Y | Y | N | Y | Y | Y | Y | Y | Y | Y | Y | Y | Y | Y | N | Y | N | Y | Y | N | Y | Y | Y | Y | Y | Y | Y |
| 36 | Sudathip et al.(36), 2019 | Y | Y | Y | N | Y | Y | Y | Y | Y | Y | Y | Y | Y | Y | Y | Y | Y | Y | N | N | N | Y | Y | N | N | Y | Y | Y |
| 37 | Wanapirak et al.(37), 2019 | Y | Y | Y | N | Y | Y | Y | Y | Y | N | Y | Y | Y | Y | Y | Y | Y | N | N | Y | N | Y | Y | Y | N | Y | Y | Y |
| 38 | Suphanchaimat et al.(38), 2020 | Y | Y | Y | N | Y | Y | Y | Y | Y | N | Y | Y | Y | Y | Y | Y | Y | N | Y | Y | N | Y | Y | Y | Y | Y | Y | Y |
| 39 | Bierhoff et al.(39), 2021 | Y | Y | Y | N | Y | Y | Y | Y | Y | N | Y | Y | NA | Y | Y | Y | Y | N | N | Y | N | Y | Y | Y | N | Y | Y | Y |
| 40 | Luangasanatip et al.(40), 2021 | Y | Y | Y | N | Y | Y | Y | Y | Y | Y | Y | Y | Y | Y | Y | Y | Y | N | N | Y | N | Y | Y | Y | N | Y | Y | Y |
| 41 | Rochanathimoke et al.(41), 2021 | Y | Y | Y | N | Y | Y | Y | Y | Y | Y | Y | Y | Y | Y | Y | Y | Y | Y | N | Y | N | Y | Y | Y | N | Y | Y | Y |
| 42 | Suphanchaimat et al.(42), 2021 | N | Y | Y | N | Y | Y | Y | Y | Y | N | Y | Y | NA | Y | Y | Y | Y | Y | Y | N | N | Y | Y | N | Y | Y | Y | Y |
| 43 | Khongmee et al.(43), 2022 | Y | Y | Y | N | Y | Y | Y | Y | Y | N | Y | Y | Y | Y | Y | NA | NA | N | N | N | N | Y | Y | N | N | Y | Y | Y |
| 44 | Nantanee et al.(44), 2022 | Y | Y | Y | N | Y | Y | Y | Y | N | N | Y | Y | Y | Y | Y | Y | Y | N | N | Y | N | Y | Y | Y | N | Y | Y | Y |
| 45 | Turongkaravee et al.(45), 2022 | Y | Y | Y | N | Y | Y | Y | Y | Y | Y | Y | Y | Y | Y | Y | Y | Y | N | Y | Y | N | Y | Y | Y | N | Y | Y | Y |
| 46 | Wang et al.(46), 2023 | Y | Y | Y | N | Y | Y | Y | Y | Y | N | Y | Y | NA | Y | Y | Y | Y | Y | Y | Y | N | Y | Y | Y | Y | Y | Y | N |
| 47 | Botwright et al.(47), 2023 | Y | Y | Y | N | Y | Y | Y | Y | Y | Y | Y | Y | Y | Y | Y | Y | Y | N | N | Y | Ⓟ | Y | Y | Y | Y | Y | Y | Y |
| 48 | Cai et al.(48), 2023 | Y | Y | Y | N | Ⓟ | Y | Y | Y | Y | Y | Y | Y | Y | Y | Y | Y | Ⓟ | Y | Ⓟ | Y | N | Y | Y | Y | N | Y | Y | Y |
| 49 | Grant et al.(49), 2023 | Y | Y | Y | N | Y | Y | N | Y | Y | Y | Y | Y | Y | Y | Y | Y | Y | N | Y | Y | N | Y | Y | Y | N | Y | Y | Y |
| 50 | Janekrongtham et al.(50), 2023 | Y | Y | Y | N | Y | Y | Y | Y | N | N | N | N | N | Y | Y | Y | Y | N | N | N | N | N | Y | N | N | Y | Y | Y |
| 51 | Kositamongkol et al.(51), 2023 | Y | Y | Y | N | Y | Y | Y | Y | Y | Y | Y | Y | Y | Y | Y | Y | Y | Y | Y | Y | Y | Y | Y | Y | Y | Y | Y | Y |
| 52 | Laopachee et al.(52), 2023 | Y | Y | Y | N | Y | Y | Y | N | N | N | N | N | N | Y | Y | N | N | N | N | Y | N | Y | Y | Y | N | Y | N | N |
| 53 | Lertwilaiwittaya et al.(53), 2023 | Y | Y | Y | N | Y | Y | Y | Y | Y | Y | Y | Y | Y | Y | Y | Y | Y | Y | N | N | Y | Y | Y | Y | N | Y | Y | Y |
| 54 | Ngamprasertchai et al.(54), 2023 | Y | Y | Y | N | Y | Y | Y | Y | Y | Y | Y | Y | Y | Y | Y | Y | Y | Y | Y | Y | N | Y | Y | Y | N | Y | Y | Y |
| 55 | Palakai et al.(55), 2023 | Y | Y | Y | N | Y | Y | Y | N | Y | N | Y | Y | Y | Y | Y | N | N | N | Y | Y | N | N | Y | Y | N | Y | Y | Y |
| 56 | Prasitwarachot et al.(56), 2023 | Y | Y | Y | N | Y | Y | Y | Y | Y | Y | Y | Y | Y | Y | Y | Y | Y | N | N | Y | N | Y | Y | Y | N | Y | Y | Y |
| 57 | Sirison et al.(57), 2023 | Y | Y | Y | N | Y | Y | Y | Y | Y | Y | Y | Y | NA | Y | N | Y | Y | Y | Y | N | N | Y | Y | N | Y | Y | Y | Y |
| 58 | Srisubat et al.(58), 2023 | Y | Y | Y | N | Y | Y | Y | Y | Y | Y | Y | Y | Y | Y | Y | Y | Y | N | Y | Y | N | Y | Y | Y | N | Y | Y | Y |
| 59 | Suthutvoravut et al.(59), 2023 | Y | Y | Y | N | Y | Y | Y | Y | Y | Y | Y | Y | NA | Y | Y | Y | Y | Y | N | N | N | Y | Y | N | N | Y | Y | Y |
| 60 | Prayoonhong et al.(60), 2024 | Y | Y | Y | N | Y | Y | Y | Y | Y | N | Y | Y | Y | Y | Y | NA | NA | N | N | Y | N | Y | Y | Y | N | Y | Y | Y |

Y: yes; **Ⓟ**: partially yes; N: No**;** NA: not assessed

# **References**

1. Thavorn K, Chaiyakunapruk N. A cost-effectiveness analysis of a community pharmacist-based smoking cessation programme in Thailand. Tob Control. 2008;17(3):177-82.

2. Tengtrisorn S, Sangsupawanitch P, Chansawang W. Cost effectiveness analysis of a visual screening program for primary school children in Thailand. J Med Assoc Thai. 2009;92(8):1050-6.

3. Chotivitayatarakorn P, Chotivitayatarakorn P, Poovorawan Y. Cost-effectiveness of rotavirus vaccination as part of the national immunization program for Thai children. Southeast Asian J Trop Med Public Health. 2010;41(1):114-25.

4. Leelukkanaveera Y, Sithisarankul P, Hirunsutthikul N. Provider-initiated HIV counseling and testing of out patients at community hospitals in Thailand: an economic evaluation using the Markov model. Asian Biomedicine. 2010;4(3):479-84.

5. Sritipsukho P, Riewpaiboon A, Chaiyawat P, Kulkantrakorn K. Cost-effectiveness analysis of home rehabilitation programs for Thai stroke patients. J Med Assoc Thai. 2010;93 Suppl 7:S262-70.

6. Lee BY, Connor DL, Kitchen SB, Bacon KM, Shah M, Brown ST, et al. Economic value of dengue vaccine in Thailand. Am J Trop Med Hyg. 2011;84(5):764-72.

7. Leelahavarong P, Teerawattananon Y, Werayingyong P, Akaleephan C, Premsri N, Namwat C, et al. Is a HIV vaccine a viable option and at what price? An economic evaluation of adding HIV vaccination into existing prevention programs in Thailand. BMC Public Health. 2011;11:534.

8. Praditsitthikorn N, Teerawattananon Y, Tantivess S, Limwattananon S, Riewpaiboon A, Chichareon S, et al. Economic evaluation of policy options for prevention and control of cervical cancer in Thailand. Pharmacoeconomics. 2011;29(9):781-806.

9. Hunchangsith P, Barendregt JJ, Vos T, Bertram M. Cost-effectiveness of various tuberculosis control strategies in Thailand. Value Health. 2012;15(1 Suppl):S50-5.

10. Khiaocharoen O, Pannarunothai S, Riewpaiboon W, Ingsrisawang L, Teerawattananon Y. Economic Evaluation of Rehabilitation Services for Inpatients with Stroke in Thailand: A Prospective Cohort Study. Value Health Reg Issues. 2012;1(1):29-35.

11. Kingkaew P, Maleewong U, Ngarmukos C, Teerawattananon Y. Evidence to inform decision makers in Thailand: a cost-effectiveness analysis of screening and treatment strategies for postmenopausal osteoporosis. Value Health. 2012;15(1 Suppl):S20-8.

12. Muangchana C, Riewpaiboon A, Jiamsiri S, Thamapornpilas P, Warinsatian P. Economic analysis for evidence-based policy-making on a national immunization program: a case of rotavirus vaccine in Thailand. Vaccine. 2012;30(18):2839-47.

13. Termrungruanglert W, Havanond P, Khemapech N, Lertmaharit S, Pongpanich S, Khorprasert C, et al. Cost and effectiveness evaluation of prophylactic HPV vaccine in developing countries. Value Health. 2012;15(1 Suppl):S29-34.

14. Ditsuwan V, Lennert Veerman J, Bertram M, Vos T. Cost-effectiveness of interventions for reducing road traffic injuries related to driving under the influence of alcohol. Value Health. 2013;16(1):23-30.

15. Kulpeng W, Leelahavarong P, Rattanavipapong W, Sornsrivichai V, Baggett HC, Meeyai A, et al. Cost-utility analysis of 10- and 13-valent pneumococcal conjugate vaccines: protection at what price in the Thai context? Vaccine. 2013;31(26):2839-47.

16. Rattanavipapong W, Koopitakkajorn T, Praditsitthikorn N, Mahasirimongkol S, Teerawattananon Y. Economic evaluation of HLA-B*15:02 screening for carbamazepine-induced severe adverse drug reactions in Thailand. Epilepsia. 2013;54(9):1628-38.

17. Saokaew S, Permsuwan U, Chaiyakunapruk N, Nathisuwan S, Sukonthasarn A, Jeanpeerapong N. Cost-effectiveness of pharmacist-participated warfarin therapy management in Thailand. Thromb Res. 2013;132(4):437-43.

18. Pattanaprateep O, Chuansumrit A, Kongsakon R. Cost-Utility Analysis of Home-Based Care for Treatment of Thai Hemophilia A and B. Value Health Reg Issues. 2014;3:73-8.

19. Sangmala P, Chaikledkaew U, Tanwandee T, Pongchareonsuk P. Economic evaluation and budget impact analysis of the surveillance program for hepatocellular carcinoma in Thai chronic hepatitis B patients. Asian Pac J Cancer Prev. 2014;15(20):8993-9004.

20. Saokaew S, Tassaneeyakul W, Maenthaisong R, Chaiyakunapruk N. Cost-effectiveness analysis of HLA-B*5801 testing in preventing allopurinol-induced SJS/TEN in Thai population. PLoS One. 2014;9(4):e94294.

21. Srisubat A, Sriratanaban J, Ngamkiatphaisan S, Tungsanga K. Original article. Cost-effectiveness of annual microalbuminuria screening in Thai diabetics. Asian Biomedicine. 2014;8(3):371-9.

22. Tozan Y, Ratanawong P, Louis VR, Kittayapong P, Wilder-Smith A. Use of insecticide-treated school uniforms for prevention of dengue in schoolchildren: a cost-effectiveness analysis. PLoS One. 2014;9(9):e108017.

23. Thiboonboon K, Leelahavarong P, Wattanasirichaigoon D, Vatanavicharn N, Wasant P, Shotelersuk V, et al. An Economic Evaluation of Neonatal Screening for Inborn Errors of Metabolism Using Tandem Mass Spectrometry in Thailand. PLoS One. 2015;10(8):e0134782.

24. Sakulsupsiri A, Sakthong P, Winit-Watjana W. Cost-Effectiveness Analysis of the Self-Management Program for Thai Patients with Metabolic Syndrome. Value Health Reg Issues. 2016;9:28-35.

25. Tosanguan J, Chaiyakunapruk N. Cost-effectiveness analysis of clinical smoking cessation interventions in Thailand. Addiction. 2016;111(2):340-50.

26. Kantito S, Saokaew S, Yamwong S, Vathesatogkit P, Katekao W, Sritara P, et al. Cost-effectiveness analysis of patient self-testing therapy of oral anticoagulation. J Thromb Thrombolysis. 2018;45(2):281-90.

27. Kittikraisak W, Suntarattiwong P, Ditsungnoen D, Pallas SE, Abimbola TO, Klungthong C, et al. Cost-effectiveness of inactivated seasonal influenza vaccination in a cohort of Thai children </=60 months of age. PLoS One. 2017;12(8):e0183391.

28. Kotirum S, Muangchana C, Techathawat S, Dilokthornsakul P, Wu DB, Chaiyakunapruk N. Economic Evaluation and Budget Impact Analysis of Vaccination against Haemophilus influenzae Type b Infection in Thailand. Front Public Health. 2017;5:289.

29. Phisalprapa P, Supakankunti S, Charatcharoenwitthaya P, Apisarnthanarak P, Charoensak A, Washirasaksiri C, et al. Cost-effectiveness analysis of ultrasonography screening for nonalcoholic fatty liver disease in metabolic syndrome patients. Medicine (Baltimore). 2017;96(17):e6585.

30. A. P, P. P, A. V. Oral Presentations. Nephrology. 2021;26(S1):3-31.

31. Kumdee C, Kulpeng W, Teerawattananon Y. Cost-utility analysis of the screening program for early oral cancer detection in Thailand. PLoS One. 2018;13(11):e0207442.

32. Marino R, Traub F, Lekfuangfu P, Niyomsilp K. Cost-effectiveness analysis of a school-based dental caries prevention program using fluoridated milk in Bangkok, Thailand. BMC Oral Health. 2018;18(1):24.

33. Dilokthornsakul P, Kengkla K, Saokaew S, Permsuwan U, Techasaensiri C, Chotpitayasunondh T, et al. An updated cost-effectiveness analysis of pneumococcal conjugate vaccine among children in Thailand. Vaccine. 2019;37(32):4551-60.

34. Phisalprapa P, Supakankunti S, Chaiyakunapruk N. Cost-effectiveness and budget impact analyses of colorectal cancer screenings in a low- and middle-income country: example from Thailand. J Med Econ. 2019;22(12):1351-61.

35. Saokaew S, Prasitsuebsai W, Bibera GL, Kengkla K, Zhang XH, Oh KB, et al. Economic Evaluation of Human Rotavirus Vaccine in Thailand. Infect Dis Ther. 2019;8(3):397-415.

36. Sudathip P, Kongkasuriyachai D, Stelmach R, Bisanzio D, Sine J, Sawang S, et al. The Investment Case for Malaria Elimination in Thailand: A Cost-Benefit Analysis. Am J Trop Med Hyg. 2019;100(6):1445-53.

37. Wanapirak C, Buddhawongsa P, Himakalasa W, Sarnwong A, Tongsong T. Fetal Down syndrome screening models for developing countries; Part II: Cost-benefit analysis. BMC Health Serv Res. 2019;19(1):898.

38. Suphanchaimat R, Doung-Ngern P, Ploddi K, Suthachana S, Phaiyarom M, Pachanee K, et al. Cost Effectiveness and Budget Impact Analyses of Influenza Vaccination for Prisoners in Thailand: An Application of System Dynamic Modelling. Int J Environ Res Public Health. 2020;17(4).

39. Bierhoff M, Angkurawaranon C, Rijken MJ, Sriprawa K, Kobphan P, Nosten FN, et al. Tenofovir disoproxil fumarate in pregnancy for prevention of mother to child transmission of hepatitis B in a rural setting on the Thailand-Myanmar border: a cost-effectiveness analysis. BMC Pregnancy Childbirth. 2021;21(1):157.

40. Luangasanatip N, Mahikul W, Poovorawan K, Cooper BS, Lubell Y, White LJ, et al. Cost-effectiveness and budget impact analyses for the prioritisation of the four available rotavirus vaccines in the national immunisation programme in Thailand. Vaccine. 2021;39(9):1402-14.

41. Rochanathimoke O, Riewpaiboon A, Praditsitthikorn N, Tharmaphornpilas P, Jiamsiri S, Thavorncharoensap M, et al. Economic evaluation of rotavirus vaccination: an important step of the introduction to the national immunization program in Thailand. Expert Rev Pharmacoecon Outcomes Res. 2021;21(4):811-9.

42. Suphanchaimat R, Tuangratananon T, Rajatanavin N, Phaiyarom M, Jaruwanno W, Uansri S. Prioritization of the Target Population for Coronavirus Disease 2019 (COVID-19) Vaccination Program in Thailand. Int J Environ Res Public Health. 2021;18(20).

43. Khongmee T, Torpongpun A, Intarachumnum S, Kulthanachairojana N. Cost-Utility Analysis of Specialized Multidisciplinary Care Versus Standard Care in the Management of Patients with Reduced Ejection Fraction Heart Failure in Thailand. Journal of the Medical Association of Thailand. 2022;105(8):719-23.

44. Nantanee R, Sriratanaban J. Cost-effectiveness and estimated net monetary benefits of a fluoride varnish application program during well-child visits by 9- to 30-month-old children in three areas of Thailand. Community Dent Oral Epidemiol. 2023;51(3):512-8.

45. Turongkaravee S, Praditsitthikorn N, Ngamprasertchai T, Jittikoon J, Mahasirimongkol S, Sukasem C, et al. Economic Evaluation of Multiple-Pharmacogenes Testing for the Prevention of Adverse Drug Reactions in People Living with HIV. Clinicoecon Outcomes Res. 2022;14:447-63.

46. Wang Y, Luangasanatip N, Pan-Ngum W, Isaranuwatchai W, Prawjaeng J, Saralamba S, et al. Assessing the cost-effectiveness of COVID-19 vaccines in a low incidence and low mortality setting: the case of Thailand at start of the pandemic. Eur J Health Econ. 2023;24(5):735-48.

47. Botwright S, Win EM, Kapol N, Benjawan S, Teerawattananon Y. Cost-Utility Analysis of Universal Maternal Pertussis Immunisation in Thailand: A Comparison of Two Model Structures. Pharmacoeconomics. 2023;41(1):77-91.

48. Cai CGX, Lim NW, Huynh VA, Ananthakrishnan A, Dabak SV, Dickens BSL, et al. Economic Analysis of Border Control Policies during COVID-19 Pandemic: A Modelling Study to Inform Cross-Border Travel Policy between Singapore and Thailand. Int J Environ Res Public Health. 2023;20(5).

49. Grant A, Tan CJ, Wattanasirichaigoon S, Rungruanghiranya S, Thongphiew A, Thavorn K, et al. Cost-effectiveness analysis of the SMART quit clinic program in smokers with cardiovascular disease in Thailand. Tob Induc Dis. 2023;21(April):47.

50. Janekrongtham C, Punsuwan N, Thitichai P, Lertpiriyasuwat C, Pan-Ngum W, Poovorawan K, et al. Cost-effectiveness of tenofovir prophylaxis during pregnancy for the elimination of mother-to-child transmission of the hepatitis B virus: real-world analysis from Thailand. BMJ Open. 2023;13(7):e067275.

51. Kositamongkol C, Kanchanasurakit S, Mepramoon E, Talungchit P, Chaopotong P, Kengkla K, et al. Cost-utility and budget impact analyses of cervical cancer screening using self-collected samples for HPV DNA testing in Thailand. BMC Public Health. 2023;23(1):2413.

52. Laopachee P, Siripongsakun S, Sangmala P, Chanree P, Hiranrat P, Srisittimongkon S. Cost-Effectiveness Analysis of Ultrasound Surveillance for Cholangiocarcinoma in an Endemic Area of Thailand. Asian Pac J Cancer Prev. 2023;24(12):4117-25.

53. Lertwilaiwittaya P, Tantai N, Maneeon S, Kongbunrak S, Nonpanya N, Hurst ACE, et al. A cost-utility analysis of BRCA1 and BRCA2 testing in high-risk breast cancer patients and family members in Thailand: a cost-effective policy in resource-limited settings. Front Public Health. 2023;11:1257668.

54. Ngamprasertchai T, Kositamongkol C, Lawpoolsri S, Rattanaumpawan P, Luvira V, Chongtrakool P, et al. A cost-effectiveness analysis of the 13-valent pneumococcal conjugated vaccine and the 23-valent pneumococcal polysaccharide vaccine among Thai older adult. Front Public Health. 2023;11:1071117.

55. Palakai R, Sornpaisarn B, Sawangdee Y, Chuanwan S, Saonuam P, Katewongsa P, et al. The cost-effectiveness of improved brief interventions for tobacco cessation in Thailand. Front Public Health. 2023;11:1289561.

56. Prasitwarachot R, Thavorn K, Patikorn C, Wattanasirichaigoon S, Rungruanghiranya S, Thongphiew A, et al. A cost-effectiveness analysis of national smoking cessation services among chronic obstructive pulmonary disease patients in Thailand. J Med Econ. 2023;26(1):1377-85.

57. Sirison K, Nittayasoot N, Techasuwanna R, Cetthakrikul N, Suphanchaimat R. Cost-Effectiveness Analysis of COVID-19 Vaccine Booster Dose in the Thai Setting during the Period of Omicron Variant Predominance. Trop Med Infect Dis. 2023;8(2).

58. Srisubat A, Kittrongsiri K, Sangroongruangsri S, Khemvaranan C, Shreibati JB, Ching J, et al. Cost-Utility Analysis of Deep Learning and Trained Human Graders for Diabetic Retinopathy Screening in a Nationwide Program. Ophthalmol Ther. 2023;12(2):1339-57.

59. Suthutvoravut U, Kunakorntham P, Semayai A, Tansawet A, Pattanaprateep O, Piebpien P, et al. Cost-effectiveness analysis of isolation strategies for asymptomatic and mild symptom COVID-19 patients. Cost Eff Resour Alloc. 2023;21(1):85.

60. Prayoonhong W, Sonsingh W, Permsuwan U. Clinical outcomes and economic evaluation of patient-centered care system versus routine-service system for patients with type 2 diabetes in Thailand. Heliyon. 2024;10(3):e25093.
